# Dual Antibacterial Activities and Biofilm Eradication of a Marine Peptide-N6NH_2_ and Its Analogs against Multidrug-Resistant *Aeromonas veronii*

**DOI:** 10.3390/ijms21249637

**Published:** 2020-12-17

**Authors:** Ting Li, Zhenlong Wang, Huihui Han, Da Teng, Ruoyu Mao, Ya Hao, Na Yang, Xiumin Wang, Jianhua Wang

**Affiliations:** 1 Gene Engineering Laboratory, Feed Research Institute, Chinese Academy of Agricultural Sciences, Beijing 100081, China; liting7427@163.com (T.L.); wzlcqut@163.com (Z.W.); hhh05170907@163.com (H.H.); tengda@caas.cn (D.T.); maoruoyu@caas.cn (R.M.); haoya@caas.cn (Y.H.); yangna@caas.cn (N.Y.); 2 Key Laboratory of Feed Biotechnology, Ministry of Agriculture and Rural Affairs, Beijing 100081, China; 3 Chinese Herbal Medicine Laboratory, Feed Research Institute, Chinese Academy of Agricultural Sciences, Beijing 100081, China

**Keywords:** marine peptide-N6NH_2_, non-coded amino acid, *Aeromonas veronii*, biofilm, antimicrobial activity

## Abstract

*Aeromonas veronii* is one of the main pathogens causing various diseases in humans and animals. It is currently difficult to eradicate drug-resistant *A. veronii* due to the biofilm formation by conventional antibiotic treatments. In this study, a marine peptide-N6NH_2_ and its analogs were generated by introducing Orn or replacing with D-amino acids, Val and Pro; their enzymic stability and antibacterial/antibiofilm ability against multi-drug resistant (MDR) *A. veronii* ACCC61732 were detected in vitro and in vivo, respectively. The results showed that DN6NH_2_ more rapidly killed *A. veronii* ACCC61732 and had higher stability in trypsin, simulated gastric/intestinal fluid, proteinase K, and mouse serum than the parent peptide-N6NH_2_. DN6NH_2_ and other analogs significantly improved the ability of N6NH_2_ to penetrate the outer membrane of *A. veronii* ACCC61732. DN6NH_2_, N6PNH_2_ and V112N6NH_2_ protected mice from catheter-associated biofilm infection with MDR *A. veronii* ACCC61732, superior to N6NH_2_ and CIP. DN6NH_2_ had more potent efficacy at a dose of 5 μmol/kg (100% survival) in a mouse peritonitis model than other analogs (50–66.67%) and CIP (83.33%), and it inhibited the bacterial translocation, downregulated pro-inflammatory cytokines, upregulated the anti-inflammatory cytokine, and ameliorated multiple-organ injuries (including the liver, spleen, lung, and kidney). These data suggest that the analogs of N6NH_2_ may be a candidate for novel antimicrobial and antibiofilm agents against MDR *A. veronii* infections.

## 1. Introduction

*Aeromonas veronii* is an emerging aquatic pathogen that can cause diarrhea, wound infections, and hemorrhagic septicemia in humans and animals, including aquatic animals with 40–60% cumulative mortality [1,2]. In recent years, there has been an increasing number of cases of large-scale *A. veronii* outbreaks. It has been reported that *A. veronii* can infect freshwater fish [3,4], amphibians [5], and mammals [6], resulting in serious economic losses to the aquaculture industry and threatening food safety. In addition, people can infect through contacting with *A. veronii*-contaminated surfaces, such as contaminated livestock and poultry meat and seafood, resulting in gastroenteritis [7], wound infections [8], and other diseases in the elderly and children with low immunity [9]. *A. veronii* can form biofilms; aggregates of bacteria are embedded within a self-produced matrix of extracellular substances [10], which further complicates the treatment because biofilm-encased bacteria can be 10–1000-fold more tolerant to conventional antibiotics than their planktonic counterparts [11]. Approximately 96% of *Aeromonas* isolates from humans and water conditions are resistant to ampicillin, tetracycline, ciprofloxacin (CIP), chloramphenicol, tetracycline, cotrimoxazole, etc. [12,13]. Therefore, there is an urgent and growing need for the development of alternative antimicrobial agents with superior properties and less toxicity for the prevention and treatment of *A. veronii* infections.

Antimicrobial peptides (AMPs), also known as host defense peptides, are natural or synthetic peptides with antimicrobial activity against diverse pathogenic bacteria in the planktonic state and/or in biofilms [14]. Some cationic AMPs and their synthetic derivatives such as LL-37, PR-39, pexiganan, temporin A, and PXL01 have been proposed to be a promising alternative for the treatment of infections caused by antibiotic-resistant bacteria [15,16]. However, major obstacles to their success as therapeutics in clinical trials are their inherent susceptibility to proteolytic degradation, high toxicity to eukaryotic cells, or low stability under physiologically relevant conditions in vivo (e.g., in the presence of proteases (trypsin and chymotrypsin), salt, and serum) [17,18]. To overcome these problems, many researchers have tried to develop novel AMPs with improved stability and selective toxicity by several strategies, including the use of non-coded amino acids (D-amino acids, aminoisobutyric acid, etc.), chemical modification (N-acetylation, C-amidation, cyclization, etc.), and non-peptidic backbones of peptides [19,20]. Among them, the use of ornithine (Orn) as a charged moiety is preferable to the use of a non-coded amino acid, and it provides the stability of AMPs such as Api88, Api137, and Apidaecin against proteases [21,22,23,24]. Unfortunately, some modifications such as N-acetylation and D-enantiomer can improve the stability of AMPs towards proteases, accompanied by unfavorable effects on antibacterial activity or some toxicity towards eukaryotic cells [19].

In our previous studies, C-terminal amidated marine peptide-N6 (N6NH_2_) displayed potent intracellular and extracellular antibacterial activity against a variety of bacteria, especially Gram-negative bacteria including *Escherichia* and *Salmonella* strains [25]. However, N6NH_2_ is very sensitive to trypsin, which greatly limits its clinical application in the future. In this study, various modified peptides of N6NH_2_ were designed by using natural residues (Val and Pro), unnatural residue (Orn), and D-amino acids for the first time, aiming to improve its stability towards proteases and antibacterial activity, while keeping the structural features (β-sheet) known to be important for the antimicrobial activity of AMPs. The extracellular/intracellular antibacterial activity and anti-biofilm ability of N6NH_2_ and its analogs were tested against multidrug-resistant (MDR) *A. veronii*, as well as their stability, toxicity, resistance, and mechanisms of action. Additionally, the efficacy of all analogs was determined in a mouse model of catheter-associated biofilm infection and in a peritonitis mouse model of bacterial infection with MDR *A. veronii*.

## 2. Results

### 2.1. Design and Physicochemical Properties of N6NH_2_ and Its Analogs

To develop marine peptides with high stability, low toxicity, and high efficacy, four new analogs of N6NH_2_ were designed based on the following criteria: (a) keeping the structural features (β-sheet) that are crucial for effective antibacterial activity of N6NH_2_; (b) changing net charge and hydrophobicity; and (c) replacing residues with natural amino acids (Pro and Val) and non-natural ones (D-enantiomers and Orn). N6PNH_2_ and V112N6NH_2_ were generated by the substitution of Arg19 and Gly1,12 in the sequences of N6NH_2_ with Pro and Val, respectively. DN6NH_2_ and Guo-N6NH_2_ were produced by using D-enantiomers of all amino acids (in the exception of Gly) and Orn in N6NH_2_, respectively (Table 1). 

The chemical structures of four analogs were similar to those of the parent N6NH_2_ (Figures S1 and S2), indicating the structural features (β-sheet) of N6NH_2_ were maintained after modification. The physicochemical properties (including pI, net charge, and hydrophobicity) of DN6NH_2_ (+5) are equal to those of N6NH_2_ (Table 1). The hydrophobicity of N6PNH_2_ and V112N6NH_2_ are higher than that of N6NH_2_. Both V112N6NH_2_ and N6NH_2_ have the same net charges (+5). Guo-N6NH_2_ (+6) has more positive charges than N6NH_2_ (+5) and N6PNH_2_ (+4), indicating their different antibacterial activity; more net charges of AMPs may facilitate the initial electrostatic attraction of peptides with negatively charged components on the bacterial membrane surface.

### 2.2. N6NH_2_ and Its Analogs Showed Potent Antimicrobial Activity

The minimum inhibitory concentration (MIC) values of N6NH_2_ against *A. veronii*, *Escherichia coli*, and *Salmonella* were 1.61–12.90, 0.81–1.61, and 0.81–3.23 μM, respectively, which were slightly lower than those of DN6NH_2_ (1.62–25.86, 1.62–3.23, and 0.81–6.46 μM) (Table 2). The activity of Guo-N6NH_2_ (3.04–12.16, 0.76–1.52, and 0.76–3.04 μM) was almost identical to that of N6NH_2_. However, N6PNH_2_ (6.62–26.49, 1.66–3.31, and 0.83–13.25 μΜ) and V112N6NH_2_ (6.25–12.51, 1.56–3.13, 0.78–6.25 μM) have higher MIC values, which indicated their lower antibacterial activity than their parent N6NH_2_. The MICs of DN6NH_2_ against *Pseudomonas aeruginosa* and Gram-positive bacteria such as *Staphylococcus aureus* and *S. hyicus* were 1.62–6.46 μM, lower than those of N6NH_2_ (6.46–25.8 µΜ), indicating that DN6NH_2_ possesses antibacterial activity than N6NH_2_. Additionally, all peptides did not display any activity against *Candida albicans*.

**Figure 1:**
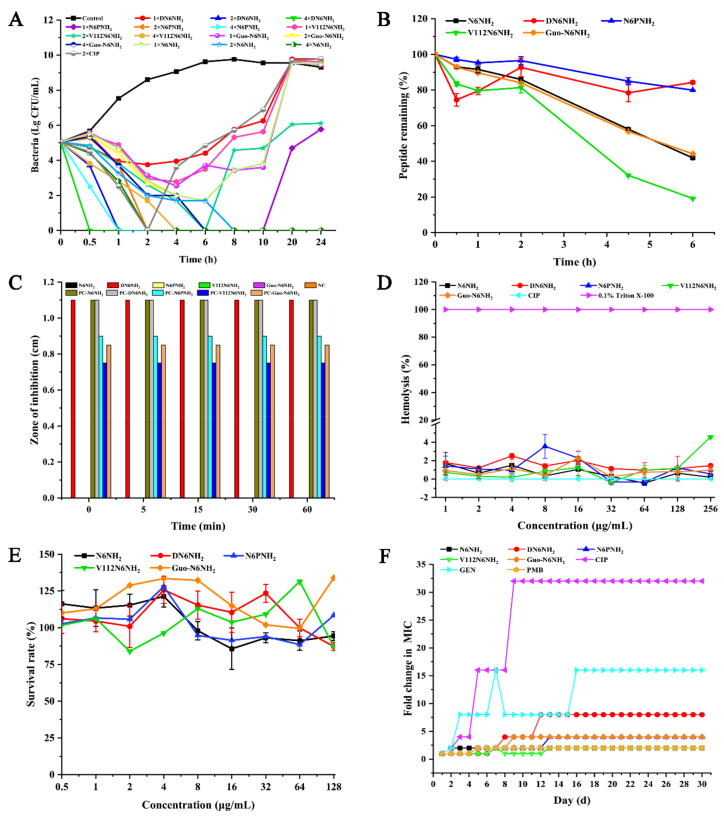
Time-killing curves, toxicity, and stability of N6NH_2_ and its analogs: (**A**) time-killing curves of N6NH_2_ and its analogs (1×, 2×, or 4× MIC) against *A. veronii* ACCC61732. CIP (2× MIC) and PBS were used as the positive and negative control, respectively; (**B**) changes of peptide content of N6NH_2_ and its analogs in serum of mouse with time; (**C**) the stability of N6NH_2_ and its analogs in SIF (NC, negative control; PC, positive control); (**D**) hemolytic activity of N6NH_2_ and its analogs or CIP at different concentrations (1–256 μg/mL) against mouse erythrocytes; (**E**) cytotoxicity of peptides or CIP at different concentrations (0.5–128 μg/mL) against *A. veronii* ACCC61732 in RAW 264.7 cells; and (**F**) resistance of N6NH_2_ and its analogs.

The bactericidal kinetics showed that 1× MIC of N6NH_2_ analogs could not completely kill *A. veronii* ACCC61732, while 2× MIC DN6NH_2_ could completely kill *A. veronii* ACCC61732 within 1 h, which is more rapid than N6NH_2_ (8 h), N6PNH_2_ (2 h), and Guo-N6NH_2_ (6 h) (Figure 1A); however, 2× MIC V112N6NH_2_ was unable completely kill *A. veronii* ACCC61732. Comparably, the bacteria treated with 2× MIC CIP showed a slow reduction and started to regrow after 2 h. The results indicate that DN6NH_2_ has the strongest bactericidal activity.

### 2.3. N6NH_2_ and Its Analogs Showed Additive Effects with Antibiotics and Greater Post-Antibiotic Effect (PAE) Values

The efficacy of interactions between peptides and traditional antibiotics against *A. veronii* ACCC61732 is shown in Tables S1–S5. The fractional inhibitory concentration index (FICI) showed that the activity of N6NH_2_ combined with rifampicin, streptomycin sulfate, doxycycline hyclate, and kanamycin sulfate was 16–25-fold greater than that of N6NH_2_ alone. Furthermore, N6NH_2_ showed additive effects with rifampicin, streptomycin sulfate, doxycycline hyclate, and kanamycin sulfate (Table S1). Notably, antagonism between N6NH_2_ and antibiotics was not found in the combinations. Comparably, N6NH_2_ had synergic effect with polymyxin B (PMB). Other analogs showed an additive effect with some antibiotics including norfloxacin, streptomycin sulfate, rifampicin, and PMB (Tables S2–S5). Additionally, Guo-N6NH_2_ had a synergic effect with PMB (Table S5).

The PAE results of peptides on *A. veronii* ACCC61732 are shown in Table 3. All peptides and CIP showed increased PAE values in a dose-dependent manner. The PAE values of DN6NH_2_ on *A. veronii* ACCC61732 was 3.36 h at 4× MIC, significantly greater than that of N6NH_2_ (1.17 h), N6PNH_2_ (0.68 h), V112N6NH_2_ (2.15 h), Guo-N6NH_2_ (2.07 h), and CIP (0.67 h). Meanwhile, the PAEs of V112N6NH_2_ (0.74–2.15 h) and Guo-N6NH_2_ (0.62–2.07 h) were higher than those of N6NH_2_ (0.69–1.17 h), with the exception of N6PNH_2_ (0.52–0.68 h).

### 2.4. DN6NH_2_ and N6PNH_2_ Had Higher Stability in Different Conditions

The temperature, salt, serum, enzyme, and pH stabilities of peptides were evaluated by the MIC assay (Table S6). After treatment at different temperatures (4–80 °C) for 1 h, pH values (2–10) for 3 h, and salt concentrations (50–500 mM) for 3 h, DN6NH_2_ retained its intrinsic antibacterial activity against MDR *A. veronii* ACCC61732, but its activity at 100 °C was slightly reduced. The activity of Guo-N6NH_2_, N6PNH_2_, and V112N6NH_2_ was reduced by 1–3-fold. Noticeably, Guo-N6NH_2_ had higher activity than N6PNH_2_ and V112N6NH_2_ at different salt conditions. In the enzyme stability, DN6NH_2_ retained the antibacterial activity against *A. veronii* ACCC61732 under pepsin, trypsin, and protease K conditions, but N6PNH_2_, V112N6NH_2_, and Guo-N6NH_2_ lost their activity in trypsin and protease K conditions. MS analysis of trypsin-treated N6NH_2_ and DN6NH_2_ showed that the structure of DN6NH_2_ is not affected by trypsin (Figures S3 and S4). Additionally, after incubation in the mouse serum for 2–4.5 h, 92.73–78.41% of DN6NH_2_ and 95.23–84.94% of N6PNH_2_ retained intact native patterns, higher than those of N6NH_2_ (85.89–57.98%), Guo-N6NH_2_ (84.03–56.75%), and V112N6NH_2_ (81.36–32.11%) (Figure 1B). After a 6 h-incubation in serum, the remaining rates of DN6NH_2_ and N6PNH_2_ were 84.25% and 79.94%, respectively, higher than those of N6NH_2_ (41.93%), Guo-N6NH_2_ (44.24%), and V112N6NH_2_ (19.12%). These results indicate that both DN6NH_2_ and N6PNH_2_ have higher stability in serum or enzyme conditions than N6NH_2_ and other analogs.

The susceptibility of peptides to simulated gastric fluid (SGF) and simulated intestinal fluid (SIF) was detected by using the inhibition zone. The results showed that, after treatment in SGF for 60 min, DN6NH_2_ displayed higher antibacterial activity than N6NH_2_ and other analogs (Figure 1C). Meanwhile, DN6NH_2_ retained 100% activity against *A. veronii* ACCC61732, but N6NH_2_ and other analogs were degraded in less than 5 min in SIF (Figure S5), indicating higher stability of DN6NH_2_ than N6NH_2_ in SIF.

### 2.5. All Analogs Exhibited No or Low Toxicity and Resistance

The hemolysis activities of N6NH_2_, DN6NH_2_, N6PNH_2_, V112N6NH_2_, Guo-N6NH_2_, and CIP were 0.19%, 1.46%, 0.56%, 4.59%, 0.93%, and 0%, respectively, at a concentration of 256 μg/mL, indicating that, similar to N6NH_2_, all analogs have no or very low hemolysis to mouse erythrocytes (Figure 1D).

RAW 264.7 cells were used to evaluate the toxicity of peptides. The survival rate of DN6NH_2_ was over 100% at low concentrations; the cell survival rates of N6NH_2_, DN6NH_2_, N6PNH_2_, V112N6NH_2_, and Guo-N6NH_2_ were 94.39%, 87.26%, 108.44%, 87.18%, and 133.93%, respectively, at a concentration of 128 μg/mL, indicating that all peptides have no or very low toxic effects on animals (Figure 1E).

After 30 serial passages of *A. veronii* ACCC61732 in the presence of peptides, the MICs of N6NH_2_, DN6NH_2_, N6PNH_2_, V112N6NH_2_, and Guo-N6NH_2_ were increased by one-fold, seven-fold, three-fold, one-fold, and three-fold, respectively, indicating that DN6NH_2_, N6PNH_2_, and Guo-N6NH_2_ can induce very low bacterial resistances within 30 days (Figure 1F). In contrast, CIP and gentamicin (GEN) significantly induced bacterial resistances with the MIC increased by 15-fold and 31-fold, respectively, after 30 passages. The MIC of PMB was enhanced by one-fold, indicating that, similar to PMB, N6NH_2_ and its analogs have lower resistance than CIP and GEN, which may serve as novel antibacterial agents against MDR *A. veronii* ACCC61732.

### 2.6. Antibacterial Mechanism of N6NH_2_ and Its Analogs

#### 2.6.1. N6NH_2_ and Its Analogs Permeabilized the Outer Cell Membranes and Disrupted the Membrane Potentials

**Figure 2:**
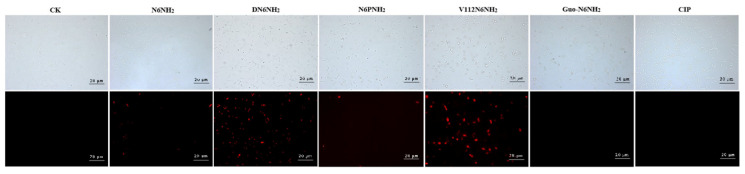
Fluorescence inverted microscope analysis of N6NH_2_ and its analogs against *A. veronii* ACCC61732 membrane penetrating ability. The magnification of images is 20 μm.

The ability of peptides to permeabilize the bacterial outer membranes was measured by N-phenyl-1-naphthylamine (NPN) probe staining. As shown in Figure 2 and Figure S6A, all peptides induced a time- and concentration-dependent increase in fluorescence. The addition of 1× and 2× MIC of peptides to *A. veronii* ACCC61732 cells caused a slow increase in fluorescence intensity, indicating a weak outer membrane permeability of peptides. However, 4× MIC of peptides could permeabilize *A. veronii* ACCC61732 within 1 min and fluorescence was increased by one-fold, indicating that all peptides can instantly permeabilize the outer membrane of *A. veronii* cells. DN6NH_2_, V112N6NH_2_, Guo-N6NH_2_, and PMB have a higher ability to penetrate the outer membrane than N6NH_2_, while V112N6NH_2_ has the best ability to penetrate the outer membrane of *A. veronii* ACCC61732.

In the absence of peptides, 100% of cells exhibited no propidium iodide (PI) staining, indicating the intact cell membranes (Figure 2 and Figure S8). After treatment with 4× MIC DN6NH_2_, the membrane permeabilizing ratio of bacteria increased to 41.93% (0.5 h) and 33.73% (2 h). After treatment with 4× MIC V112N6NH_2_ for 0.5–2 h, the penetration ratio increased up to 4.28% and 23.93%, respectively, higher than that of N6NH_2_ (0–1.12%) (Figure S8 and Table S7), indicating that both DN6NH_2_ and V112N6NH_2_ have stronger ability to destroy the integrity of the cell membrane of *A. veronii* ACCC61732 than their parent N6NH_2_. However, N6NH_2_, N6PNH_2_, Guo-N6NH_2_, and CIP have poor ability to destroy the cell membrane integrity of *A. veronii* ACCC61732.

After treatment of *A. veronii* ACCC61732 with peptides, the inner membrane potential changes are shown in Figure S6B–F. In 3,3′-dipropylthiadicarbocyanine iodide DiSC3(5) analysis, the fluorescence intensity of DiSC3(5) was significantly increased by the addition of peptides. Compared with CIP, N6NH_2_ and its analogs showed a relatively strong and concentration-dependent ability to depolarize cytoplasmic bacterial membranes.

#### 2.6.2. Peptides Exposure Affects Adenosine Triphosphate (ATP) Release from Cells

ATP efflux measured by luminescent microbial cell viability assays, in contrast to CIP, indicates a rapid leakage of ATP following the addition of N6NH_2_ and its analogs to intermediate cultures of *A. veronii* ACCC61732. When incubated at 8×, 16×, and 32× MIC concentrations for 60 min, N6NH_2_ showed ATP release of 56.07–90.00%, DN6NH_2_ showed ATP release of 84.34–86.15%, N6PNH_2_ showed ATP release of 77.39–90.34%, V112N6NH_2_ showed ATP release of 88.14–90.34%, and V112N6NH_2_ showed ATP release of 88.14–90.00%. Guo-N6NH_2_ showed ATP release of 66.23–92.09%, higher than CIP (approximately 7.06–47.60%). DN6NH_2_ was also found to induce ATP leakage in bacteria even under low MIC conditions (Figure S6G). The results indicate that N6NH_2_ and its derivatives have a role in causing ATP leakage in bacteria.

#### 2.6.3. All Analogs of N6NH_2_ Bound to Bacterial Genomic DNA and Changed DNA Structure

The interaction between DNA and peptides is shown in Figure S7A. The DNA-binding ability of all peptides was enhanced with the increased mass ratio of peptide-DNA. When the mass ratios were up to 10.0, DN6NH_2_ completely inhibited the migration of genomic DNA, more potently than its parent and other analogs. However, CIP could not inhibit the migration of genomic DNA. This result indicates that similar to N6NH_2_, all analogs can bind to genomic DNA.

The circular dichroism (CD) spectra were used to further confirm the DNA-binding affinity of peptides. As presented in Figure S7B, the CD spectra of *A. veronii* ACCC61732 genomic DNA showed a positive peak at 270 nm and a negative peak at 245 nm in the absence of peptides. After exposure to DN6NH_2_, the elliptic intensity of the positive band declined with the increased peptide concentration, suggesting that DN6NH_2_ may insert into base pairs of DNA and weakened stacking interaction. However, the peak positions of positive and negative peaks did not change and shift in the presence of other analogs of N6NH_2_, indicating that N6NH_2_, N6PNH_2_, V112N6NH_2_, and Guo-N6NH_2_ do not affect the double helix of genomic DNA.

### 2.7. All Analogs of N6NH_2_ Induced Morphological Changes in A. veronii

#### 2.7.1. Scanning Electron Microscope (SEM) Observations

**Figure 3:**
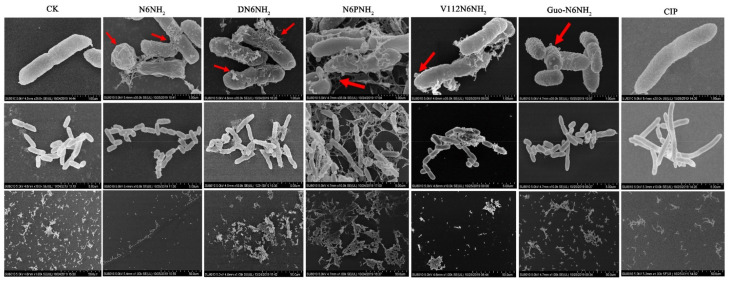
SEM images of *A. veronii* ACCC61732 cells treated with N6NH_2_ and its analogs. Bacteria in mid-logarithmic growth were treated with peptides or antibiotic at 4× MIC or CIP for 2 h (*n* = 3 independent experiments). CIP was used as the positive control. Red arrows indicate typical disruptions, which were caused by peptides or CIP (filamentous substances, OMVs, protrusions, and leakage of contents).

The untreated *A. veronii* ACCC61732 cells exhibited intact smooth surfaces, but the cells treated with peptides underwent considerable morphological changes (Figure 3). After treatment with DN6NH_2_, over 50% cells produced many filamentous substances and the collapsed cells were observed. After treatment with N6NH_2_, N6PNH_2_, and V112N6NH_2_, it appeared to numerous protrusions or blebs and filamentous substances outside of the cells. After treatment with V112N6NH_2_ and Guo-N6NH_2_, numerous protruding outer membrane vesicles (OMVs) were seen in the *A. veronii* ACCC61732 cells (Figure 3). After polymerase chain reaction (PCR) amplification with the primers of the outer membrane vesicle gene-*vacJ* (Table S8), the product (482 bp) was obtained in gel (Figure S9), indicating that the *vac*J ABC (ATP-binding cassette) transport system may be involved in *A. veronii* ACCC61732 OMVs formation [26]. In contrast, the *A. veronii* ACCC61732 cells treated with CIP could not be divided normally and formed longer rod-shaped bacteria. These results suggest that there are different mechanisms of action between peptides and CIP.

#### 2.7.2. Transmission Electron Microscope (TEM) and Confocal Laser Scanning Microscope (CLSM) Observations

**Figure 4:**
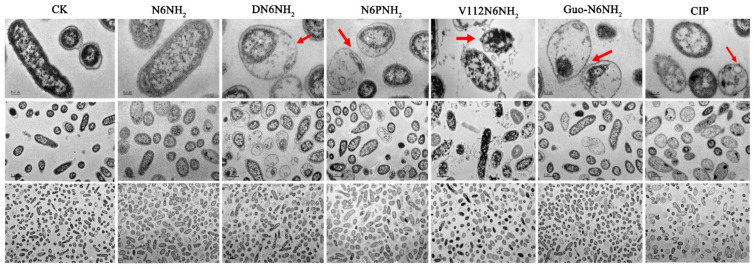
TEM images of *A. veronii* ACCC61732 cells treated with N6NH_2_ and its analogs. Bacteria in mid-logarithmic growth were treated with peptides or antibiotic at 4× MIC or CIP for 2 h (*n* = 3 independent experiments). CIP was used as the positive control. Red arrows indicate typical disruptions, which were caused by peptides or CIP (filamentous substances, disappearance of membranes, leakage of contents, and ghosts). The scale of the top, middle and bottom images is 0.2, 1.0, and 2.0 μm, respectively.

Effects of peptides on *A. veronii* ACCC61732 cells were also visualized using TEM. The untreated *A. veronii* ACCC61732 cells displayed normal morphology, intact cell membranes, and homogeneous electron density in cytoplasm. After treatment with 4× MIC peptides or CIP for 2 h, heterogeneous electron density, disappearance of the outer and inner cell membranes, leakage of cellular contents, and ghosts were observed in *A. veronii* ACCC61732 cells, which is a typical feature of cell death. DN6NH_2_, N6PNH_2_, Guo-N6NH_2_, and CIP induced more abnormal cells than N6NH_2_ (Figure 4). However, noticeably, after exposure to V112N6NH_2_, many OMVs were observed on the surface of the bacterial cell membrane (Figure 4 and Figure S10), which may be related to the host immune regulation and the promotion of bacterial survival during envelope stress [27].

### 2.8. N6NH_2_ and Its Analogs Eliminated MDR A. veronii Biofilms and Persisters

#### 2.8.1. Inhibition of Biofilm Formation

**Figure 5:**
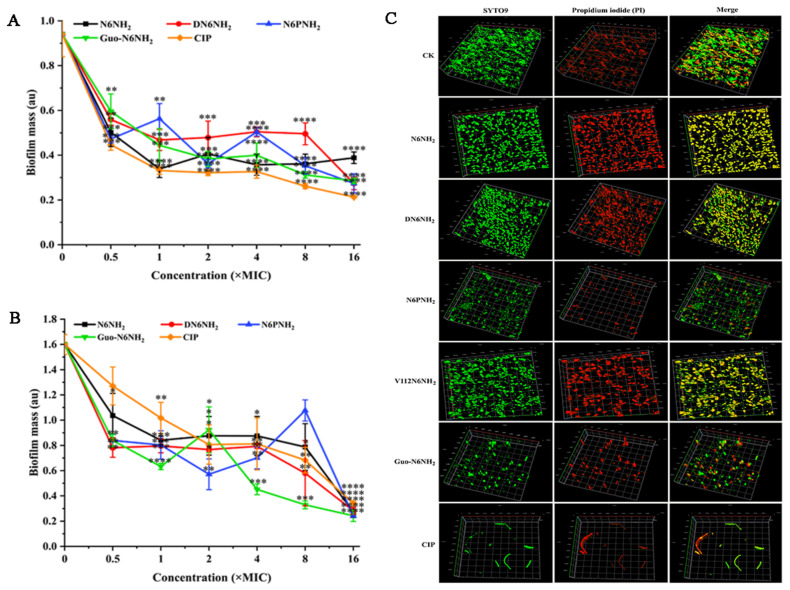
Effects of N6NH_2_ and its analogs on biofilms of *A. veronii* ACCC61732: (**A**) effect of N6NH_2_ and its analogs on early biofilms of *A. veronii* ACCC61732; (**B**) effect of N6NH_2_ and its analogs on mature biofilms of *A. veronii* ACCC61732; (**C**) laser confocal observation of the effect of N6NH_2_ and its analogs on mature biofilm of *A. veronii* ACCC61732. The analyses were measured by one-way ANOVA, with Duncan’s multiple comparisons test. A *p*-value of <0.05 was considered significant. (*) Indicates the significance between control and treatment groups. * *p* < 0.05; ** *p* < 0.01, *** *p* < 0.001, **** *p* < 0.0001.

To investigate the inhibitory effect of peptides on early biofilms, *A. veronii* ACCC61732 cells were exposed to different concentrations of peptides or CIP. After treatment with 16× MIC peptides, the biofilm of *A. veronii* ACCC61732 was inhibited by 57.59% (N6NH_2_), 71.33% (DN6NH_2_), 67.49% (N6PNH_2_), and 65.09% (Guo-N6NH_2_), respectively, indicating that DN6NH_2_ is more effective in preventing the formation of early biofilms. After treatment with 16× MIC CIP, the biofilm of *A. veronii* ACCC61732 was reduced by 70.75% (Figure 5A). Differently, however, *A. veronii* ACCC61732 treated with V112N6NH_2_ produced a large amount of precipitated substances, which may be related to the formation of OMVs. Therefore, it is difficult to evaluate the inhibitory effect of V112N6NH_2_ on early biofilms of *A. veronii* ACCC61732 by crystal violet staining. These data suggest that DN6NH_2_, N6PNH_2_, Guo-N6NH_2_, and CIP (with the exception of V112N6NH_2_) have a more potent ability to inhibit early biofilm formation than N6NH_2_.

#### 2.8.2. Eradication of Mature Biofilms

To determine the destructive potential of peptides to mature biofilms of MDR *A. veronii* ACCC61732, the bacteria were incubated for 24 h in advance. As shown in Figure 5B,C and Figure S11, the untreated bacterial cells formed thick biofilms on the surfaces of the glass plates; conversely, DN6NH_2_, N6PNH_2_, Guo-N6NH_2_, and CIP (in the exception of V112N6NH_2_) more potently inhibited *A. veronii* biofilm formation than N6NH_2_. Specifically, after treatment with 16× MIC peptides, *A. veronii* ACCC61732 biofilms were inhibited by 91.57% (N6NH_2_), 91.90% (DN6NH_2_), 97.16% (N6PNH_2_), and 97.04% (Guo-N6NH_2_). After treatment with 16× MIC CIP, the biofilm was inhibited by 89.87%. Similarly, the crystal violet staining method cannot be used to evaluate the effects of V112N6NH_2_ on mature biofilms of *A. veronii* ACCC61732. These data suggest that DN6NH_2_, N6PNH_2_, and Guo-N6NH_2_ have stronger destructive abilities on mature biofilms than N6NH_2_ and CIP.

#### 2.8.3. Killing Persisters in Biofilm

**Figure 6:**
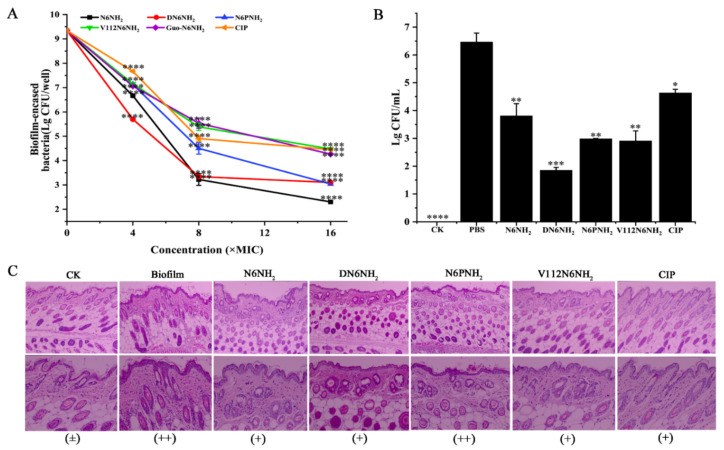
Effects of N6NH_2_ and its analogs on biofilm in mice: (**A**) effects of N6NH_2_ and its analogs on retained bacteria in early biofilms; (**B**) effects of N6NH_2_ and its analogs on biofilm formation in mouse; (**C**) effects of N6NH_2_ and its analogs on skin injuries induced by *A. veronii* ACCC61732 biofilm. Catheters with *A. veronii* ACCC61732 biofilm were incubated in the skin of the backs of mice treated with N6NH_2_ and its analogs (5 μmol/kg). Skins were harvested and detected at 7 d post infection. (**Top**) Microscope magnification 100 times; (**Bottom**) microscope magnification 200 times. The analyses were measured by one-way ANOVA, with Duncan’s multiple comparisons test. A *p*-value of <0.05 was considered significant. (*) Indicates the significance between control and treatment groups. * *p* < 0.05; ** *p* < 0.01, *** *p* < 0.001, **** *p* < 0.0001.

MDR *A. veronii* significantly reduced after treatment with peptides; 8× MIC DN6NH_2_, N6PNH_2_, V112N6NH_2_, Guo-N6NH_2_, N6NH_2_ and CIP killed 64.22%, 57.99%, 46.21%, 46.26%, 65.49%, and 47.46% of *A. veronii* ACCC61732, respectively. Meanwhile, 66.79%, 67.44%, 52.04%, 54.31%, 75.36%, and 52.32% of bacteria cells were killed by 16× MIC DN6NH_2_, N6PNH_2_, V112N6NH_2_, Guo-N6NH_2_, N6NH_2_, and CIP, respectively (Figure 6A). This indicates that N6NH_2_, DN6NH_2_, N6PNH_2_, V112N6NH_2_, Guo-N6NH_2_, and CIP can kill bacteria in *A. veronii* biofilm in a concentration-dependent manner, which is consistent with the results of the biofilm inhibition.

### 2.9. N6NH_2_ and Its Analogs Protected Mice from Catheter-Associated Biofilm Infection with MDR A. veronii

#### 2.9.1. Protection of Biofilm-Infected Mice

In a mouse model of catheter-associated biofilm infection, the mice were injected with 5 μmol/kg peptides or CIP at 24 h after catheter implantation with MDR *A. veronii* ACCC61732 biofilms. After treatment with 5 μmol/kg DN6NH_2_, N6PNH_2_, and V112N6NH_2_, the bacterial cells in the catheter were significantly decreased by 71.55%, 54.09%, and 55.29%, respectively, higher than those of N6NH_2_ (41.48%) and CIP (28.72%) (Figure 6B). Guo-N6NH_2_ was excluded due to its toxic effect on mice. This indicates that DN6NH_2_, N6PNH_2_, and V112N6NH_2_ exhibit more potent ability to inhibit the biofilm formation and alleviate abscesses in vivo than N6NH_2_ and CIP.

#### 2.9.2. Protection of Tissues from *A. veronii* ACCC61732 Biofilm

To investigate whether peptides (excluding Guo-N6NH_2_) protect mice from *A. veronii* ACCC61732 biofilm-induced skin injury, the skin was examined at seven days after treatment with peptides. There was no abnormality in the mouse skin tissue in the blank control, indicating that the implantation of sterile catheters does not affect the mouse skin; however, in the negative control, infiltration of lymphocytes in the epidermis and obvious inflammation were observed in the local area, indicating that *A. veronii* ACCC61732 biofilms can severely damage the mouse skin tissues. In contrast, after treatment with 5 μmol/kg peptides (excluding Guo-N6NH_2_) or CIP, the swelling and injury of skin tissues were apparently alleviated and no obvious pathological changes were found in the mice at seven days, suggesting that N6NH_2_ and its analogs (excluding Guo-N6NH_2_) or CIP improve the *A. veronii* ACCC61732 biofilm-induced skin damage in mice (Figure 6C). The efficacy of DN6NH_2_ and V112N6NH_2_ is superior to N6PNH_2_ and CIP, but slightly inferior to N6NH_2_.

### 2.10. N6NH_2_ and Its Analogs Protected Mice from Bacterial Infection with MDR A. veronii

#### 2.10.1. Protection of Mice

**Figure 7:**
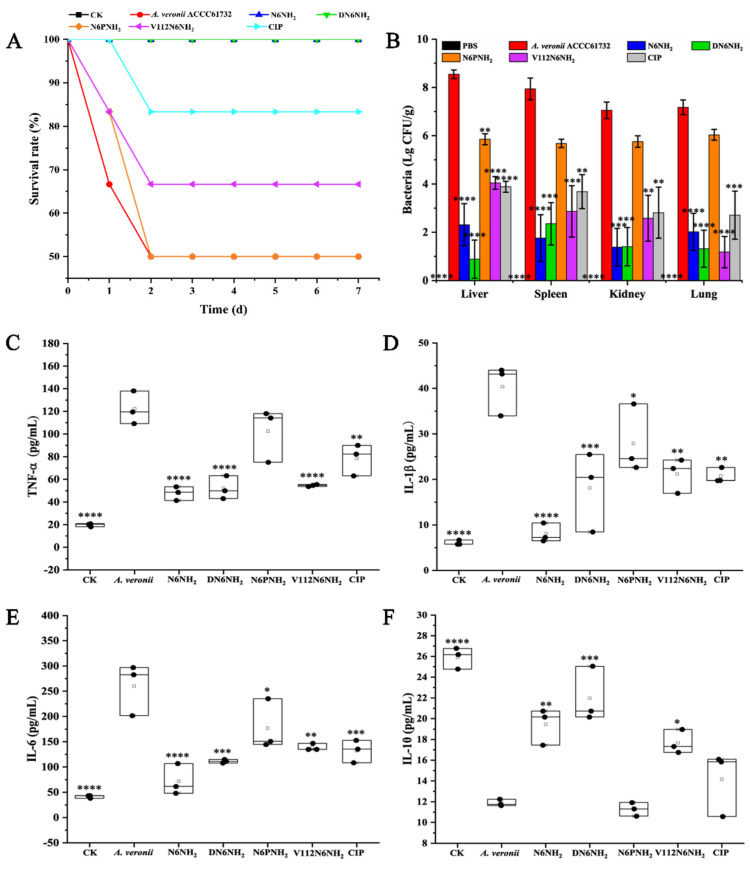
Efficacy of N6NH_2_ and its analogs in a mouse peritonitis model infected with *A. veronii* ACCC61732. (**A**) Survival of mice. Mice were infected intraperitoneally with *A. veroni* ACCC61732 (6 × 10^8^ CFU/mL, 200 μL) and treated with N6NH_2_, DN6NH_2_, N6PNH_2_, and V112N6NH_2_ (5 μmol/kg) or CIP (1 μmol/kg) after 0.5 h post infection. Survival was recorded for seven days; (**B**) the bacterial counts of mice in livers, spleens, kidneys, and lungs after treatment with peptides (5 μmol/kg) or CIP (1 μmol/kg). The untreated mice were used as the negative control. Data are expressed as mean ± standard error of mean (*n* = 6); (**C**–**F**) effects on sera cytokines. Mice were challenged with *A. veronii* ACCC61732 (6 × 10^8^ CFU/mL, 200 μL) followed by injection with peptides (5 μmol/kg) or CIP (1 μmol/kg). Sera were collected and the levels of TNF-α (**C**), IL-1β (**D**), IL-6 (**E**), and IL-10 (**F**) were detected by using an ELISA kit 24 h after treatment. Each black circle represents data from a single mouse. The analyses were measured by one-way ANOVA, with Duncan’s multiple comparisons test. A *p*-value of <0.05 was considered significant. (*) Indicates the significance between control and treatment groups. * *p* < 0.05; ** *p* < 0.01, *** *p* < 0.001, **** *p* < 0.0001.

In the peritonitis model, the mice were injected with peptides (excluding Guo-N6NH_2_) (5 μmol/kg) or CIP (1 μmol/kg) at 0.5 h after infection with MDR *A. veronii* ACCC61732 (6 × 10^8^ CFU/mL, 200 μL). The untreated mice began to die at 24 h after inoculation with *A. veronii* ACCC61732, and 50% died within 48 h. After treatment with 5 μmol/kg N6PNH_2_ and V112N6NH_2_, the survival rates of the mice were 50% and 66.67%, respectively. All mice survived when treated with 5 μmol/kg N6NH_2_ and DN6NH_2_. The survival rate of mice treated with 1 μmol/kg CIP was 83.33% (Figure 7A). The mice in the blank control survived throughout the experimental period, indicating that the therapeutic efficiency of N6NH_2_ and DN6NH_2_ in peritonitis mice is greater than that of N6PNH_2_, V112N6NH_2_, and CIP.

#### 2.10.2. Inhibition of Bacterial Translocation

To test whether *A. veronii* ACCC61732 translocate from the peritoneal cavity to other deep organs, livers, spleens, kidneys, and lungs were harvested and homogenized at 24 h post-treatment with peptides (excluding Guo-N6NH_2_) or CIP; bacteria in tissue homogenates were counted. As shown in Figure 7B, bacterial counts (Lg CFU/g) in the livers, spleens, kidneys, and lungs of the untreated mice were 8.55, 7.94, 7.05, and 7.18, respectively. After treatment with 5 μmol/kg N6NH_2_, *A. veronii* ACCC61732 cells were significantly reduced in livers (72.94%), spleens (77.86%), kidneys (80.52%), and lungs (71.89%); 5 μmol/kg DN6NH_2_ reduced the bacterial burden in livers (89.65%), spleens (70.38%), kidneys (80.10%), and lungs (81.61%); after treatment with 5 μmol/kg N6PNH_2_, *A. veronii* ACCC61732 cells reduced in livers (31.54%), spleens (28.47%), kidneys (18.36%), and lungs (15.92%); and after treatment with 5 μmol/kg V112N6NH_2_, *A. veronii* ACCC61732 cells decreased in livers (52.71%), spleens (63.87%), kidneys (63.42%), and lungs (83.57%). Nevertheless, in the CIP-treated mice (1 μmol/kg), the bacterial counts were decreased in livers (54.55%), spleens (53.62%), kidneys (60.14%), and lung (62.27%), indicating that DN6NH_2_ (with the exception of spleens) has higher activity than N6NH_2_ and CIP. Both N6PNH_2_ and V112N6NH_2_ have intermediate therapeutic effects, with N6PNH_2_ being worse.

#### 2.10.3. Regulation of Cytokines

To explore whether effects of peptides (excluding Guo-N6NH_2_) and CIP on the protection are associated with cytokines, the serum levels of pro-inflammatory (tumor necrosis factor-α (TNF-α), interleukin-1β (IL-1β), and interleukin-6 (IL-6)) and anti-inflammatory cytokine (interleukin-10 (IL-10)) were determined by the enzyme-linked immunosorbent assay (ELISA) kit. After 24 h treatment with N6NH_2_, the levels of TNF-α, IL-1β, IL-6, and IL-10 in mice were 40.65–55.67, 6.06–10.82, 47.22–108.59, and 17.02–20.87 pg/mL, respectively; after treatment with DN6NH_2_, the levels of TNF-α, IL-1β, IL-6, and IL-10 in mice were 40.02–66.20, 8.17–25.94, 104.48–115.43, and 15.94–25.46 pg/mL, respectively; after treatment with V112N6NH_2_, the levels of TNF-α, IL-1β, IL-6, and IL-10 in the mice were 52.05–59.01, 16.41–24.86, 133.08–150.00, and 16.48–19.60 pg/mL, respectively (Figure 7C–F). The levels of TNF-α, IL-1β, and IL-6 in the N6NH_2_- and DN6NH_2_-treated groups were remarkably lower than those of the untreated control (108.36–140.31, 29.63–44.87, and 200.48–304.48 pg/mL, respectively). The levels of IL-10 in N6NH_2_- and DN6NH_2_-treated mice were significantly greater than those of the untreated control (11.08–12.67 pg/mL) and CIP-treated groups (10.1–16.41 pg/mL). However, there was no discrepancy of IL-10 levels between the N6PNH_2_- or CIP-treated mice and untreated mice, indicating that N6PNH_2_ and CIP have poor ability to modulate immune factors. Similar to N6NH_2_ and CIP, DN6NH_2_ and V112N6NH_2_ can inhibit the production of proinflammatory cytokines and upregulate the anti-inflammatory cytokine level.

#### 2.10.4. Alleviation of the Organ Injury

**Figure 8:**
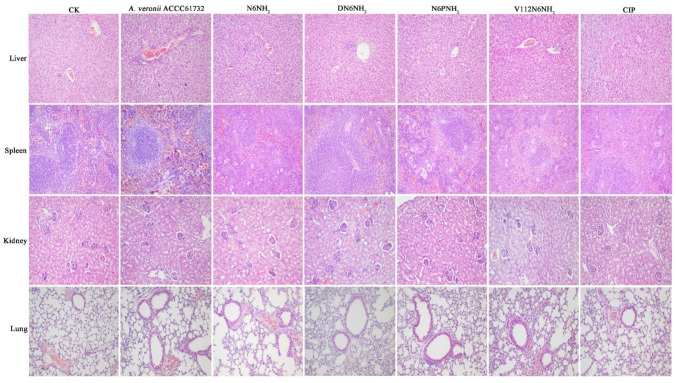
Effects of N6NH_2_ and its analogs on organ injury in mice. Mice were infected intraperitoneally with *A. veronii* ACCC61732 (6 × 10^8^ CFU/mL, 200 μL) and treated with N6NH_2_, DN6NH_2_, N6PNH_2_, and V112N6NH_2_ (5 μmol/kg) or CIP (1 μmol/kg). Livers, spleens, kidneys, and lungs were harvested from mice sacrificed at five days after infection. The magnification of images is 200 times.

No pathological symptom was observed in the liver, spleen, kidney, and lung of the uninfected mice (Figure 8). In the untreated (24 h after infection) control, the liver tissue was severely damaged, including swollen and deformed liver cells, necrotic foci, necrotic debris, and eosinophilic bodies; many liver lobules were inflamed or degenerated. The veins in the red pulp area were dilated and congested, and the splenic sinus was dilated and congested. Scattered infiltration of inflammatory cells was found in the local renal interstitial tissue; renal tubular atrophy and degeneration could also be seen at local inflammatory sites. Lung tissue structure was blurred; most of the inflammatory cells around the blood vessels and the bronchi and bronchioles infiltrated, and fibroblasts proliferated heavily. In contrast, the livers and spleens were apparently less damaged in the DN6NH_2_- and N6NH_2_-treated mice, and no obvious pathological changes occurred in the lungs and kidneys. N6NH_2_ and DN6NH_2_ remarkably alleviated the injury of the liver and spleen. In the mice treated with V112N6NH_2_, livers, spleens, kidneys, and lungs were significantly less damaged, but it was slightly less effective than N6NH_2_ and DN6NH_2_. In the CIP-treated mice, there was a weak therapeutic effect on livers, spleens, kidneys, and lungs, but it was superior to V112N6NH_2_. N6PNH_2_ had the worst therapeutic effect with obvious inflammation in each tissue (Figure 8). The overall efficacy of peptides at five days was superior to that at 24 h (Figure S12). These results indicate that DN6NH_2_ and N6NH_2_ improve the tissue damage or injury in mice induced by *A. veronii* ACCC61732. In summary, the efficacy of peptides may be ranked from good to bad as follows: N6NH_2_ = DN6NH_2_ > V112N6NH_2_ > CIP > N6PNH_2_.

## 3. Discussion

*A. veronii* is a widely distributed novel pathogen that can infect humans and animals. Due to overuse of broad-spectrum antibiotics in fish hatcheries, agriculture, and clinical settings, there has been an increase in antibiotic resistance of *Aeromonas* species [28], illustrating an emerging potential health concern [29]. Some marine AMPs such as N2, N6, A6, and G6 with low toxicity are highly effective against Gram-negative bacteria in vitro and in vivo and exhibit different antimicrobial mechanisms (including penetrating cell membrane and binding to genomic DNA), which open the door to the exploration of marine peptides [30,31]. In our previous study, the structure–activity relationship revealed that the analogs of N6 (including N2143, N2413, SN1, and SN3) without the structural features (β-sheet) lost the antimicrobial activity against Gram-negative bacteria (data not shown), indicating that the structural feature β-sheet is very important for the antimicrobial activity of marine peptides. Thus, in this study, to improve the stability and activity of peptide, four new modified analogs of marine peptide-N6NH_2_ in keeping the structural feature (β-sheet) were designed by changing charge and hydrophobicity based on natural or unnatural amino acids, namely Val, Pro, Orn, and D-ones, followed by the determination of antibacterial and anti-biofilm activity in vitro and in vivo for the first time.

Introduction of non-natural amino acids, especially Orn and D-amino acids, into the sequence of AMPs or substitution with some natural amino acids is an effective strategy to improve activity and prevent peptides from proteolytic degradation [19,23]. In our study, an addition of basic Orn at the N-terminus of N6NH_2_ did not improve the stability towards trypsin, serum, and SIF but slightly improved the activity against MDR *A. veronii* ACCC61732 compared to its parent N6NH_2_ (Figure 1B,C and Table 2), which is consistent with a previous report that found the activity of Api88 against *E. coli* and *Klebsiella pneumoniae* was enhanced by 1-fold and 127-fold, respectively, which may be attributed to the addition of Orn at its N-terminus [19]. The basic residue-Orn can increase positive net charges of peptides and most likely allow stronger electrostatic interactions with the negatively charged bacterial surface [19,32]. It has been reported that D-magainin, polybia-CP, and relevant derivatives may be of significant therapeutic potential due to their being highly resistant to proteolysis and nearly identical antibacterial activity [23,24]. Peptides containing D-amino acids might possess biological properties, which are similar to those of the respective natural L-enantiomer [24]. In our study, after replacement with D-residues, the antibacterial activity of DN6NH_2_ showed higher activity against some Gram-positive bacteria such as *S. aureus* and *S. hyicus*, indicating stronger broad-spectrum antibacterial activity than N6NH_2_ (Table 2). Compared with other analogs of N6NH_2_, DN6NH_2_ is more effective under physiological conditions (25% mouse serum, trypsin, and SIF) (Figure 1B,C, Figure S5, and Table S6). DN6NH_2_ remained intact even after 1 h of incubation in SIF, while all-L-enantiomer (N6NH_2_) was rapidly degraded (within 5 min) (Figure 1C). Similarly, the MIC value of DN6NH_2_ against *A. veronii* ACCC61732 was not changed (4 μg/mL) after 3 h incubation with trypsin, but N6NH_2_ lost its activity against *A. veronii* ACCC61732 (>128 μg/mL) (Table S6), which is consistent with magainin [24]. Moreover, PAE of DN6NH_2_ was greater than N6NH_2_ and other analogs (Table 3). DN6NH_2_ also enhanced the cell membrane penetrating ability and cytoplasmic membrane potential of *A. veronii* (Figure S6). More importantly, DN6NH_2_ and other analogs of N6NH_2_ induced lower resistance to *A. veronii* ACCC61732 (1–7-fold) than CIP (15-fold) and GEN (31-fold) after 30 passages (Figure 1F), which may be related to their rapid membrane permeabilizing mechanism at high concentrations of peptides (Figure 2 and Figure S6). For this reason, it is likely that more effective permeabilization of bacterial outer membrane by peptides than CIP may overcome intrinsic resistance pathways [33]. Furthermore, in the mouse peritonitis model, both N6NH_2_ and DN6NH_2_ were highly efficient in a mouse model of intraperitoneal infection, with 100% survival rates at doses of 5 μmol/kg, indicating its higher therapeutic effect than CIP and other analogs (Figure 7). The data further support our view that DN6NH_2_ can be a new potential antimicrobial candidate against *A. veronii* infections due to its very low toxicity and hemolysis (Figure 1D,E).

Biofilm is considered a biological barrier, formed by bacteria that continuously divide and multiply on biological materials through substances such as extracellular polysaccharide complexes; it is a major virulence factor contributing to the chronicity of infections and one of the most important reasons for the failure of current anti-infective therapies [34,35]. In recent years, there are increasing reports about *A. veronii*-infected aquatic animals and humans, but few studies focus on *A. veronii* biofilms [35,36]. In this study, N6NH_2_ and its analogs could effectively inhibit the early and mature biofilm formation of *A. veronii* ACCC61732 and killed persisters in biofilms in vitro and in a mouse model of catheter-associated biofilm infection (Figure 5 and Figure 6). Noticeably, DN6NH_2_, N6PNH_2_, and Guo-N6NH_2_ exhibited more potent ability to eradicate biofilms and kill persisters in a concentration-dependent manner than N6NH_2_ and CIP, indicating these analogs are potential therapeutic agents.

An interesting characteristic was the formation of OMVs at the outer membrane of *A. veronii* after treatment with V112N6NH_2_ (Figure S10), which was similar to the previous reports that sub-MIC Api peptides induced OMVs at the cell membrane of *E. coli* and *P. aeruginosa* [32]. Particularly, for membrane permeabilizing peptides, Gram-negative bacteria may form OMVs to remove membrane-interacting drug molecules, even if the membrane is not the final target of these peptides [32]. Additionally, OMVs may be related to the overexpressed or misfolded proteins in the periplasmic space, which can trigger similar bacterial cell vesicle formations, including the outer membrane and periplasmic components, and thus help bacteria remove toxic compounds from cell surfaces [32]. Overall, 4× MIC V112N6NH_2_ could more rapidly permeabilize the outer membrane of *A. veronii* within 1 min than other analogs of N6NH_2_ (Figure S6A), and it interacted with intracellular genomic DNA (Figure S7).

In conclusion, N6NH_2_ and its analogs exhibited potent antibacterial activity against *A. veronii*, with low toxicity and no or low resistance. These analogs displayed different antimicrobial mechanisms, including the permeabilization of cell membranes, interaction with genomic DNA, and formation of OMVs on the cell surfaces. The *A. veronii* biofilms and persisters were eradicated or killed by DN6NH_2_, N6PNH_2_, and V112N6NH_2_ in vitro and in mice, which is superior to N6NH_2_ and CIP. DN6NH_2_ displayed higher stability to trypsin and serum and higher efficacy at a dose of 5 μmol/kg (100% survival) in a mouse peritonitis model than other peptides and CIP (83.33%); it also inhibited bacterial translocation, downregulated cytokines, and alleviated multiple-organ injuries. This provides a new guideline to design novel antibacterial and antibiofilm marine peptides in clinical applications.

## 4. Materials and Methods

### 4.1. Reagents, Cell Lines, and Model Animals

Mueller–Hinton Broth (MHB), Mueller–Hinton Agar (MHA), Tryptic Soy Broth (TSB), and Tryptic Soy Agar (TSA) were obtained from AoBoX (Shanghai, China); PI was purchased from Sigma (Shanghai, China). The whole bacterial genome extraction kit was purchased from Tiangen Biotechnology Co., Ltd. (Beijing, China,); all antibiotics were purchased from China Veterinary Drug Supervision Institute (Beijing, China). RAW 264.7 macrophages were obtained from Peking Union Medical College (Beijing, China). Furthermore, six-week old specific-pathogen-free (SPF) female ICR mice (approximately 20 g/mouse) were purchased from the Beijing Vital River Laboratory Animal Technology Co. Ltd (Beijing, China). All tests for the evaluation of microbial and animal cells were carried out in Class II biological safety cabinets. Other chemical reagents were of analytical grade. All peptides were synthesized by Mimotopes (Wuxi, China) and their purity was greater than 90%.

### 4.2. Physiochemical Properties of N6NH_2_ and Its Analogs

The major structural parameters of peptides, including MW, pI, and net charge were calculated by ProtParam (https://web.expasy.org/protparam/). Hydrophobicity was predicated by the HeliQuest analysis website (http://heliquest.ipmc.cnrs.fr/cgi-bin/ComputParamsV2.py).

### 4.3. Antibacterial Activities and Time-Killing Curves of N6NH_2_ and Its Analogs

The MIC values of peptides and CIP were determined by the microtiter broth dilution method [37]. Briefly, the tested strains including Gram-negative bacteria, Gram-positive bacteria, and fungi were cultured to the logarithmic growth phase, diluted to 10^5^ CFU/mL, and added into 96-well plates (90 μL/well). Two-fold serial dilutions of peptides or antibiotics dissolved in PBS were added to each well (10 μL/well). CIP and PBS were used as the positive and negative control, respectively. The plates were incubated at 37 °C for 16–18 h until visible turbidity was observed in the negative control. The lowest concentration that can completely inhibit bacterial growth is the MIC value of the peptides or antibiotic against the tested strains. All experiments were repeated three times.

Time-killing curves were performed as previously described [38]. Briefly, *A. veronii* ACCC61732 was cultivated to mid-log phase at 37 °C (250 rpm), diluted to 1 × 10^5^ CFU/mL, and incubated with peptides or antibiotic (1×, 2×, and 4× MIC). The sample of 100 μL was taken from each flask at 0, 0.5, 1, 2, 4, 6, 8, 10, 20, and 24 h, respectively, and serially diluted for colony counting. PBS and CIP were used as the negative and positive control, respectively.

### 4.4. Synergism and PAE of N6NH_2_ and Its Analogs against A. veronii

The checkerboard microtiter assay was used to determine the combinations of peptides with different antibiotics (including quinolone, rifamycin, peptide antibiotics, aminoglycoside, tetracycline, and chloramphenicol). Briefly, logarithmic *A. veronii* ACCC61732 (10^5^ CFU/mL) was added into the 96-well plates; 25 μL peptides (from 1/32× to 4× MIC) were added into the 96-well plates, followed by antibiotics. The fractional inhibitory concentration (FIC) was used to calculate the combination effects using the following formula: FIC index (FICI) = MIC_(peptide in combination)_/MIC_(peptide alone)_ + MIC_(antibiotic in combination)_/MIC_(antibiotic alone)_. FICI ≤ 0.5 means synergistic, 0.5 < FICI ≤ 1 means additive action, 1 < FICI ≤ 4 means indifference, and FICI > 4 means antagonism [39].

After treatment with N6NH_2_, N6NH_2_ analogs, and CIP (1×, 2×, and 4× MIC) for 2 h, *A. veronii* ACCC61732 cells (1 × 10^8^ CFU/mL) were diluted 1000 times by medium, transferred to new flasks, and incubated at 37 °C and 250 rpm. The samples were taken from flasks for counting each hour until bacterial cultures became turbid. The untreated bacteria severed as controls. The PAE was calculated using the following equation: PAE = T − C, where T is the time (h) required for the CFU in the test culture to increase by 10-fold from the count immediately after the drug removal and C is the corresponding time (h) for the control [40]. The experiment was performed in triplicate.

### 4.5. Stability of N6NH_2_ and Its Analogs

#### 4.5.1. Temperature, pH, Salt, and Enzyme Sensitivity

The thermal stability of peptides was determined after treatment for 1 h at different temperatures (4, 20, 40, 60, 80, and 100 °C). The relative antibacterial activity of peptides against *A. veronii* ACCC61732 was determined by the MIC assay [41].

To evaluate pH stability, peptides were dissolved in glycine-HCl buffer (pH 2.0), sodium acetate buffer (pH 4.0), sodium phosphate buffer (pH 6.0), tris-HCl buffer (pH 8.0), or glycine-NaOH buffer (pH 10.0) and treated for 3 h. The MIC values of peptides against *A. veronii* ACCC61732 were measured as described above.

Similarly, salt stability of peptides was determined after a 3 h-incubation in different NaCl solutions (50, 100, 200, 300, 400, and 500 mM). Additionally, N6NH_2_ and N6NH_2_ analogs were mixed with pepsin (3000 U/mg, pH 2.0), trypsin (250 U/mg, pH 8.0), and proteinase K (40 mAU/mg, pH 7.0) solutions at a ratio of 10:1 (*w*/*w*) to evaluate the protease stability [42]. The activity of peptides against *A. veronii* ACCC61732 was determined as described above. The untreated peptides were used as the positive control and buffers alone were used as the negative control. All assays were conducted in triplicate.

#### 4.5.2. Stability in Gastric/Intestinal Fluid and Serum

The stability of N6NH_2_ and its analogs in SGF and SIF was carried out as previously described [43]. Briefly, a final concentration of 200 μg/mL peptide was incubated in SGF and SIF at 37 °C. At different time intervals, an aliquot of 20 μL mixture was taken and the activity of peptides was tested against *A. veronii* ACCC61732 by the inhibition zone assay. *A. veronii* ACCC61732 (200 μg/mL) in PBS was used as the positive control; SGF and SIF were used as negative controls.

Peptides were dissolved in mouse serum at 37 °C to detect their serum stability and samples were removed at different time to determine their residual activity by reverse-phase high-performance liquid chromatography (RP-HPLC) [30]. All assays were conducted in triplicate.

### 4.6. Hemolysis, Cytotoxicity, and Resistance of N6NH_2_ and Its Analogs

#### 4.6.1. Hemolysis

To determine the hemolysis of peptides, eyeball blood was collected from 6-week-old SPF ICR mice. The blood was centrifuged at 4 °C for 10 min (1500 rpm), washed three times with 0.9% physiological saline, and diluted to 8% suspension. The peptides were dissolved in 0.9% physiological saline (1–256 μg/mL). Red blood cell suspensions and peptide solutions (100 μL) were mixed in 96-well plates, centrifuged at 4 °C for 5 min (1500 rpm), and incubated at 37 °C for 1 h. The supernatant was added into a 96-well plate and the UV absorbance was measured at 540 nm using a microplate reader. Physiological saline and 0.1% Triton X-100 were used as 0% and 100% hemolytic controls, respectively. The hemolytic ratio was calculated as follows: Hemolysis (%) = [(Abs_peptide_ − Abs_saline_)/(Abs_0.1%Triton X-100_ − Abs_saline_)] × 100% [38].

#### 4.6.2. Cytotoxicity

To determine the cytotoxicity of peptides, RAW 264.7 cells (2.5 × 10^4^ cells/well) were seeded into 96-well plates and incubated for 24 h. A serial concentration of peptides (0.5–128 μg/mL) were added into each well and incubated for another 24 h; supernatants were removed from the wells and washed with PBS twice. The cells were dyed with 3-(4,5-dimethyl-2-thiazolyl)-2,5-diphenyl-2-H-tetrazolium bromide (MTT) (5 mg/mL) for 4 h, incubated with dimethyl sulfoxide (DMSO), and measured at 570 nm. PBS was used as controls. The cell survival rate was calculated by the equation: Survival rate (%) = (Abs_peptide/_Abs_PBS_) × 100 [25].

#### 4.6.3. Resistance

The development of bacterial resistance of peptides was performed by the MIC assays. The mid-log phase of *A. veronii* ACCC61732 (1 × 10^5^ CFU/mL) (90 μL/well) was added to a 10 μL fresh medium, containing 64×, 32×, 16×, 8×, 4×, 2×, 1×, 0.5×, 0.25×, 0.125×, and 0.0625× MIC of peptides. GEN, CIP, and PMB were used as controls. After 16–18 h incubation at 37 °C, cells from the second highest concentration, which shows visible growth, were used to inoculate the subsequent culture. The MIC values were measured as described above. The serial passaging was repeated for 30 days [30].

### 4.7. Mechanism of N6NH_2_ and Its Analogs

#### 4.7.1. Effects on the Cell Membrane and Membrane Potential

The outer membrane permeabilization abilities of peptides were determined using the fluorescent NPN assay. Mid-log phase *A. veronii* ACCC61732 was collected by centrifugation, washed twice, and then suspended in N-2-hydroxyethylpiperazine-n-2-ethane sulfonic acid (HEPES) buffer (pH 7.4) to an OD_600nm_ = 0.4. Cell suspensions and NPN solutions (10 μM) were added into the 96-well black plates, followed by the addition of peptide solutions (1×, 2×, and 4× MIC). Fluorescence intensity was recorded until no further increase with a microplate reader (excitation/emission, 328/438 nm). The cells treated with PMB and CIP were used as positive controls; *A. veronii* ACCC61732 treated with PBS was used as the negative control; the untreated *A. veronii* ACCC61732 cells were used as the blank control [44].

To analyze effects of peptides on the inner membrane, *A. veronii* ACCC61732 cells were cultured to mid-log phase at 37 °C in MHB medium, washed thrice with PBS, and resuspended in the PBS buffer to 1 × 10^8^ CFU/mL. The cells were then incubated with 1×, 2×, and 4× MIC peptides or CIP at 37 °C for 0.5 and 2 h, respectively. After fixation with PI, the samples were detected by a fluorescent inverted microscope and FACS Calibur Flow Cytometer (BD, San Jose, CA, USA), respectively. Cell Quest Pro software (BD, San Jose, CA, USA) was used for the data analysis [22,45].

The DISC3(5)assay [46] was employed to investigate cytoplasmic membrane depolarization. Cultured *A. veronii* ACCC61732 cells were washed in 5 mM HEPES buffer (pH 7.2) containing 20 mM glucose and resuspended in buffer (5 mM HEPES buffer, 20 mM glucose, and 100 mM KCl, pH 7.2) to an OD_600_ of 0.1. The uptake and self-quench of DISC3(5) dye into bacterial cells was monitored for 13 min. Peptide solutions were added and the fluorescence intensity (λexc = 620 nm, λem = 670 nm) was measured using a spectro fluorophotometer. PBS and 2% Triton X-100 were used as the blank and positive control, respectively.

#### 4.7.2. Measure of ATP Release

*A. veronii* ACCC61732 cells (1 × 10^5^ CFU/mL mid-log phase in MHB) were exposed to various concentrationds (1/2×, 2×, 4×, 8×, 16×, and 32× MIC) of N6NH_2_ (MIC 4 μg/mL), DN6NH_2_ (MIC 4 μg/mL), N6PNH_2_ (MIC 16 μg/mL), V112N6NH_2_ (MIC 16 μg/mL), Guo-N6NH_2_ (MIC 8 μg/mL), and CIP (MIC 0.125 μg/mL) for 60 min. ATP was measured using the BacTiter-GloTM Microbial Cell Viability Assay (Promega) following the manufacturer’s instructions. The assay was performed for three biological replicates at 37 °C, with luminescence recorded using a Tecan Infinite M1000 Pro plate reader. The fold reduction of ATP was calculated as: Fold reduction ATP = 1 − (L_treat_ − L_media_/L_control_ − L_media_), where L_treat_ is the luminescence of treated cells, L_media_ is the luminescence of MHB without cells, and L_control_ is the luminescence of untreated cells. Resultant graphs show mean (*n* = 3) and SEM for each data point, prepared in Prism 7 [46].

#### 4.7.3. Effects on Bacterial Genomic DNA

The gel retardation assay was used to determine the ability of peptides to bind to bacterial genomic DNA. The genomic DNA was extracted from the *A. veronii* ACCC61732 strain by using a bacterial DNA Kit (TIANGEN Biotech Co., Ltd., Beijing, China). N6NH_2_ and its analogs were dissolved in DNA-binding buffer. The samples containing peptides and genomic DNA (0.8 µg) in 20 µL DNA binding buffer were incubated at 37 °C for 10 min. The peptide/DNA ratios ranged from 0.0 to 10.0 (*w*/*w*). After electrophoresis, genomic DNA was analyzed using a Geliance 200 imaging system (PerkinElmer, Waltham, MA, USA) [37,44].

CD spectra were performed to examine the secondary structure of genomic DNA from *A. veronii* ACCC61732 after incubation with peptides. N6NH_2_ and its analogs were incubated with genomic DNA at mass ratios of 2.5 and 10.0 for 10 min at room temperature. The mixtures were loaded into a cuvette of 1.0-mm path length and scanned from 190 to 330 nm at 25 °C using a Pistar π-180 CD spectrometer (Applied Photophysics Ltd., Surry, UK) [37].

#### 4.7.4. Effects of N6NH_2_ and Its Analogs on Bacterial Morphology

MDR *A. veronii* ACCC61732 cells were cultured in MHB at 37 °C (250 rpm) until the mid-log phase of growth, harvested by centrifugation, and diluted with 0.01 M PBS (pH 7.4) to 0.5–5 × 10^8^ CFU/mL. Bacterial cells were incubated with 4× MIC peptides or CIP at 37 °C for 2 h. For SEM, the samples were harvested (5000× *g*, 5 min) and fixed with 2.5% glutaraldehyde at 4 °C overnight. The cells were dehydrated for 10 min in a graded ethanol series (50%, 70%, 90%, and 100%) and transferred to a mixture of 100% ethanol, tertiary butanol, and absolute tertiary butanol (*v*:*v* = 1:1) for 15 min. Finally, the specimens were dehydrated in a critical point dryer with liquid CO_2_, coated with gold–palladium, and observed using a QUANTA200 (FEI, Philips, Amsterdam, Netherlands) [47].

For TEM, after treatment with peptides and a series of ethanol solutions (50%, 70%, 90%, and 100%) for 8 min, the samples were transferred to a mixture (*v*:*v* = 1:1) of 100% ethanol, acetone, and absolute acetone for 15 min. Subsequently, the specimens were transferred to 1:1 mixture of absolute acetone and resin for 30 min and then to absolute epoxy resin overnight. Finally, the specimens were stained with uranyl acetate and lead citrate and observed using a JEM1400 (JEDL, Tokyo, Japan) [40].

### 4.8. Effects of N6NH_2_ and Its Analogs on Biofilms and Persisters of MDR A. veronii

#### 4.8.1. Early and Mature Biofilms

Mid-log phase *A. veronii* ACCC61732 cells (1 × 10^8^ CFU/mL, 180 µL) were added into the 96-well plates. Peptides (0.5–16× MIC, 20 μL) were added into the plates and incubated for 24 h. Effects of peptides on biofilms formation was evaluated by the crystal violet staining as previously described [48,49]. The untreated bacteria were used as the negative control (A) and fresh TSB medium was used as the blank control (A_0_). The inhibition effect of peptides on early biofilms was determined by the following equation: Biofilms (%) = [(A_peptides_ − A_0_)/(A − A_0_)] × 100.

*A. veronii* ACCC61732 cells (1 × 10^8^ CFU/mL) were cultured in TSB medium in the 96-well plates at 37 °C for 24 h. A series of concentrations of peptides (0.5–16× MIC) were added into the plates and cultured for another 24 h to form mature biofilms. The plates were dyed by crystal violet and effects of peptides or CIP on mature biofilms was determined as described above.

The logarithmic phase of *A. veronii* ACCC61732 (1 × 10^8^ CFU/mL) was added into the orifice plate; each orifice plate was placed in a sterile guide piece to biofilm attached. Then, 16× MIC of peptides or CIP were added and incubated for 24 h; the guide piece was taken out, flushed with PBS buffer for three times to remove planktonic bacteria, and fixed by 2.5% glutaraldehyde overnight at 4 °C. The cells were then dehydrated for 10 min in a graded ethanol series (50%, 70%, 90%, and 100%) and transferred to a mixture (*v*:*v* = 1:1) of 100% ethanol, tertiary butanol, and absolute tertiary butanol for 15 min. Finally, the specimens were dehydrated in a critical point dryer with liquid CO_2_, coated with gold–palladium and observed using a QUANTA200 (FEI, Philips, Amsterdam, Netherlands) [50].

*A. veronii* ACCC61732 cells (1 × 10^8^ CFU/mL) were seeded in a confocal dish and incubated for 24 h; 16× MIC peptides were added into the dish and continued to incubate for another 24 h. The untreated bacteria were used as a control. Planktonic bacteria were gently rinsed twice, and the biofilms were dyed with SYTO9 and PI (LIVE/DEAD Bac Light Bacterial Viability Kit) for 15 min. After washing with PBS, the biofilms were observed by Zeiss LSM880 confocal microscope (CLSM) (Zeiss, Oberkochen, Germany) [48,51].

#### 4.8.2. Persisters in Biofilms

The mid-log phase *A. veronii* ACCC61732 cells (1 × 10^8^ CFU/mL) were inoculated into in the 96-well plates and cultured for 24 h at 37 °C. The plates were washed twice with PBS. Subsequently, the final concentration of 4–16× MIC peptides or CIP were added into the plates and incubated for 2 h at 37 °C. PBS was used as the negative control. The plates were treated with ultrasound for 5 min and the viable bacteria were counted on the TSA plates [48,51].

### 4.9. Efficacy of N6NH_2_ and Its Analogs in a Mouse Model of Catheter-Associated Biofilm Infection

#### 4.9.1. Effects on Catheter-Associated Biofilms

The mouse experiment was performed according to the Animal Care and Use Committee of the Feed Research Institute of Chinese Academy of Agricultural Sciences (CAAS) and approved by the Laboratory Animal Ethical Committee and its Inspection of the Feed Research Institute of CAAS (AEC-CAAS-20090609).

*A. veronii* ACCC61732 biofilms were developed on a mouse intravenous catheter 0.6 × 0.3 mm (outer diameter × inner diameter). Briefly, the catheter was cut into 1 cm segments; each piece was sterilized with 70% ethanol and then dried by air. Bacterial biofilms were developed on the catheter by placing individual segments into tubes containing 1.0 mL of a cell suspension (1 × 10^8^ CFU/mL) in TSB. After incubation for 24 h at 37 °C, colonized catheters were recovered aseptically and rinsed with PBS to remove unbound bacteria.

ICR female mice (20 ± 5 g) were anesthetized with isoflurane; the flanks were shaved and the skin was cleansed with alcohol. An 8–10-mm skin incision was made and dissected to create a subcutaneous tunnel with the 1cm segment of carrying *A. veronii* ACCC61732 biofilms catheter implanted at a distance of at least 2 cm from the incision. The incision was then covered with intact skin and closed with surgical suture line and the skin was disinfected. After 24 h of infection as an in vivo biological model, six mice in each group were injected with 5 μmol/kg peptides or CIP on the mouse back. The untreated mice were used as the negative control and the mice with sterile catheter were used as the blank control. The mice were anesthetized and sacrificed after seven days. The catheters in the back of the mice were collected and thoroughly shaken in sterile PBS. Effects of peptides or CIP on *A. veronii* ACCC61732 in mice was evaluated by the colony counting method [52].

#### 4.9.2. Effects on Skin Ulcer in the Mouse Back

Catheters with *A. veronii* ACCC61732 biofilm were implanted in the skin of the mouse backs and treated with 5 μmol/kg peptides or CIP after infection 24 h. The mouse back skin tissues were taken at seven days, washed with PBS, and placed in 4% paraformaldehyde for 24 h at 4 °C. After washing with PBS, the tissues were dehydrated by different concentrations of ethanol (75%, 85%, 90%, and 95%) and then were immersed in xylene for paraffin embedding. After sectioning, the samples were stained with hematoxylin and observed under the OLYMPUS BX43 microscope [53]. The mice challenged with *A. veronii* ACCC61732 biofilm and sterile catheter served as the negative and blank control.

### 4.10. Efficacy of N6NH_2_ and Its Analogs in a Mouse Peritonitis Model

#### 4.10.1. Survival of Mice

To establish a mouse peritonitis model, the six-week-old female ICR mice (approximately 20 g/mouse) were intraperitoneally injected with the MDR *A. veronii* ACCC61732 (6 × 10^8^ CFU/mL, 0.2 mL) strain. Therapeutic groups with six mice were treated with peptides (5 μmol/kg of body weight, 0.2 mL) and CIP (1 μmol/kg of body weight, 0.2 mL) at 0.5 h post-infection by intraperitoneal injection [31]. The mice injected with only bacteria or saline served as the negative and blank control. The mouse survival was recorded daily for seven days.

#### 4.10.2. Effects on Bacterial Translocation and Cytokines

The mice (15 mice/group) were challenged with *A. veronii* ACCC61732 (6 × 10^8^ CFU/mL, 0.2 mL) by intraperitoneal injection and treated with a single dose of peptides (5 μmol/kg) or CIP (1 μmol/kg). Sera and organs (livers, kidneys, spleens, and lungs) were collected from the mice at 24 h post-treatment. The organs were homogenized in sterile PBS to evaluate bacterial translocation by colony counting. The levels of cytokines (TNF-α, IL-1β, IL-6, and IL-10) in sera were detected using the ELISA kits. The uninfected and untreated mice served as the blank control; the infected mice treated with CIP and PBS were used as the positive and negative controls, respectively.

#### 4.10.3. Effects on the Injury of Multiple Organs

The mice were intraperitoneally injected with peptides and CIP at 0.5 h post-infection of *A. veronii* ACCC61732 as described above. Livers, kidneys, spleens, and lungs were removed from mice at 24 h and five days post treatment to evaluate the organ injury. After washing with PBS, the organs were fixed in 4% paraform-aldehyde for 24 h at 4 °C, dehydrated by a graded series of ethanol (75‒95%), and infiltrated with xylene. The organs were then embedded in paraffin wax, sectioned, and stained with hematoxylin and eosin. Finally, the tissue samples were fixed and observed by the light microscope. The CIP-treated mice served as the positive control and the PBS group served as the negative control.

### 4.11. Statistical Analysis

All data are presented as means ± standard error of mean. Statistical analyses between treatments or groups were determined using one-way analysis of variance (ANOVA) models in SAS 9.2 (SAS Institute Inc., Cary, NC, USA), followed by Dunnett’s multiple comparisons test. A *p*-value of <0.05 was considered statistically significant.

## Figures and Tables

**Table 1:** Amino acid sequence and physicochemical properties of N6NH_2_ and its analogs.

Peptides	Amino Acid Sequences	Length	Theoretical MW (Da)	Measured MW (Da)	Charge (+)	GRAVY	AI	BI (kcal mol^-1^)	pI	Hydrophobicity	AH %	Extinction Coefficient (M^−1^ cm^−1^)	
N6NH_2_	GFAWNVCVYRNGVRVCHRRAN-NH_2_	21	2476.85	2476.8	5	NP	NP	NP	11.64	0.375	NP	6970	
DN6NH_2_	GfawnvcvyrnGvrvchrran-NH_2_	21	2476.85	2474.88	5	NP	NP	NP	11.64	0.375	NP	6970	
N6PNH_2_	GFAWNVCVYRNGVRVCHRPAN-NH_2_	21	2417.78	2415.81	4	NP	NP	NP	10.91	0.457	NP	6970	
V112N6NH_2_	VFAWNVCVYRNVVRVCHRRAN-NH_2_	21	2561.01	2559.04	5	NP	NP	NP	11.64	0.491	NP	6970	
Guo-N6NH_2_	Gu-OGFAWNVCVYRNGVRVCHRRAN-NH_2_	21	NP	2631.08	6	NP	NP	NP	NP	NP	NP	NP	
N2143	VCVYRGFAWNCHRRANNGVRV	21	2477.85	2475.81	4	−0.31	64.76	2.61	10.72	0.375	23.81%	6970	
N2413	VCVYRCHRRANGFAWNNGVRV	21	2477.85	2475.81	4	−0.31	64.76	2.61	10.72	0.375	0.00%	6970	
SN1	AACCGFWNVVYRNGVRVHRRN	21	2477.85	2477.83	4	−0.31	64.76	2.61	10.72	0.375	0.00%	6970	
SN3	GFWNVVYRNGVAACCRVHRRN	21	2477.85	2477.83	4	−0.31	64.76	2.61	10.72	0.375	33.33%	6970	

**Note:** Lowercase residues represent D-amino acids. The underline represents amino acid substitution or addition compared to the N6NH_2_ sequence. Molecular weight (MW) was confirmed by mass spectroscopy (MS). Grand average of hydropathicity (GRAVY) and aliphatic index (AI) were calculated from https://web.expasy.org/protparam/. Boman index (BI) was calculated from http://aps.unmc.edu/AP/prediction/prediction_main.php. Alpha helix (AH) was calculated from http://npsa-pbil.ibcp.fr/cgi-bin/npsa_automat.pl?page=npsa_hnn.html). Theoretical MW, isoelectric point (pI) and extinction coefficient were calculated from https://pepcalc.com/. Hydrophobicity means the total hydrophobicity (sum of all residue hydrophobicity indices) divided by the number of residues, and they were calculated from http://heliquest.ipmc.cnrs.fr/cgi-bin/ComputParams.py. Gu denotes N, N, N′, N′-tetramethylguanidine and O denotes L-ornithine. NP indicates no prediction.

**Table 2:** MIC values of N6NH_2_ and its analogs.

Species and Strains	MIC											
	N6NH_2_		DN6NH_2_		N6PNH_2_		V112N6NH_2_		Guo-N6NH_2_		CIP	
	μg/mL	μM	μg/mL	μM	μg/mL	μM	μg/mL	μM	μg/mL	μM	μg/mL	μM
Gram-negative bacteria												
*Aeromonas veronii* ACCC61732 ^a^	4	1.61	4	1.62	16	6.62	16	6.25	8	3.04	0.125	0.38
*A. veronii* X-1-06909 ^b^	32	12.9	32	12.93	64	26.49	32	12.51	16	6.08	0.25	0.75
*A. veronii* CL0901 ^b^	16	6.46	32	12.93	64	26.49	32	12.51	16	6.08	0.0625	0.19
*A. veronii* ATCC35624	8	3.23	64	25.86	64	26.49	16	6.25	32	12.16	<0.0625	<0.19
*Escherichia coli* CVCC195	4	1.61	8	3.23	8	3.31	8	3.13	4	1.52	<0.0625	<0.19
*E. coli* CVCC1515	2	0.81	4	1.62	4	1.66	4	1.56	2	0.76	<0.0625	<0.19
*E. coli* CVCC25922	4	1.61	8	3.23	8	3.31	8	3.13	4	1.52	<0.0625	<0.19
*E. coli* CVCCO157	4	1.61	4	1.62	8	3.31	8	3.13	4	1.52	<0.0625	<0.19
*Salmonella typhimurium* ATCC14028	4	1.61	8	3.23	16	6.62	16	6.25	4	1.52	0.0625	0.19
*S. pullorum* CVCC1809	4	1.61	8	3.23	8	3.31	8	3.13	4	1.52	<0.0625	<0.19
*S. pullorum* CVCC1789	8	3.23	16	6.46	16	6.62	16	6.25	8	3.04	<0.0625	<0.19
*S. pullorum* CVCC533	4	1.61	16	6.46	32	13.25	16	6.25	8	3.04	<0.0625	<0.19
*S. enteritidis* CVCC3377	2	0.81	2	0.81	2	0.83	2	0.78	2	0.76	<0.0625	<0.19
*Pseudomonas aeruginosa* CICC21630	64	25.8	8	3.23	>64	>26.49	32	12.5	>64	>24.3	0.125	0.38
Gram-positive bacteria												
*Staphylococcus aureus* ATCC43300	16	6.46	4	1.62	>64	>26.49	32	12.5	16	6.08	0.25	0.75
*S. aureus* ATCC546	16	6.46	16	6.46	>64	>26.49	16	6.25	16	6.08	0.125	0.38
*S. aureus* ATCC25923	32	12.9	8	3.23	>64	>26.49	32	12.5	32	12.16	0.125	0.38
*S. hyicus* NCTC10350	32	12.9	8	3.23	>128	>52.98	64	25.01	64	24.32	0.125	0.38
*S. hyicus* 437-2 ^c^	32	12.9	8	3.23	>64	>26.49	32	12.5	32	12.16	8	24.14
Fungus												
*Candida albicans* CMCC98001	>128	>52	128	51.72	>128	>52.98	>128	>50.02	>128	>48.6	>128	>386.3

**Note:** a Clinical isolated strain from the liver tissue of California sea bass in the Nankou experimental base of the Chinese Academy of Agricultural Sciences. The MDR *A. veronii* ACCC61732 strain is resistant to imipenem, meropenem, tetracycline, and trimethoprim, and it was highly sensitive to quinolone antibiotics such as ciprofloxacin, norfloxacin, and oxaliplatin. Data are representative of three independent experiments. b *A. veronii* X-1-06909 and *A. veronii* CL0901 were kindly provided by the Beijing Fisheries Research Institute. c Clinical isolated strain of Tianjin pig farm. ACCC, Agricultural Culture Collection of China; CVCC, China Veterinary Culture Collection Center; ATCC, American Type Culture Collection; CICC, China Center of Industrial Culture Collection; NCTC, National Collection of Type Cultures; CMCC, National Center for Medical Culture Collection.

**Table 3:** PAE analysis of N6NH_2_ and its analogs on *A. veronii* ACCC61732.

Peptides	PAE (h)		
	1× MIC	2× MIC	4× MIC
N6NH_2_	0.69 ± 0.01	0.7 ± 0.01	1.17 ± 0.22
DN6NH_2_	0.68 ± 0.01	1.13 ± 0.11	3.36 ± 0.19
N6PNH_2_	0.52 ± 0.01	0.62 ± 0.01	0.68 ± 0.01
V112N6NH_2_	0.74 ± 0.02	1.48 ± 0.05	2.15 ± 0.13
Guo-N6NH_2_	0.62 ± 0.01	1.32 ± 0.08	2.07 ± 0.08
CIP	0.6 ± 0.01	0.63 ± 0.1	0.67 ± 0.02

## Supplementary Materials

The following are available online at https://www.mdpi.com/1422-0067/21/24/9637/s1.

## Abbreviations

CIP	ciprofloxacin
AMP	antimicrobial peptide(s)
Orn	ornithine
MDR	multidrug resistant
MIC	minimum inhibitory concentration
MW	molecular weight
MS	mass spectroscopy
GRAVY	grand average of hydropathicity
AI	aliphatic index
BI	boman index
pI	isoelectric point
FICI	fractional inhibitory concentration index
PAE	postantibiotic effect
MTT	methylthiazolyldiphenyl-tetrazolium
SGF	simulated gastric fluid
SIF	simulated intestinal fluid
GEN	gentamicin
NPN	n-phenyl-1-naphthylamine
PI	propidium iodide
DISC3(5)^)^	3,3′-dipropylthiadicarbocyanine iodide
ATP	adenosine triphosphate
CD	circular dichroism
SEM	scanning electron microscope
OMVs	outer membrane vesicles
PCR	polymerase chain reaction
TEM	transmission electron microscope
CLSM	confocal laser scanning microscope
TNF	tumor necrosis factor
IL	interleukin
ELISA	enzyme-linked immunosorbent assay
HEPES	n-2-hydroxyethylpiperazine-n-2-ethane sulfonic acid
