# Supplementary material for: Dual Antibacterial Activities and Biofilm Eradication of a Marine Peptide-N6NH2 and Its Analogs against Multidrug-Resistant Aeromonas veronii
Source: Int J Mol Sci. 2020 Dec 17;21(24):9637. doi: 10.3390/ijms21249637 (PMC7767178; doi:10.3390/ijms21249637)
Supplement: Supplementary file 1 [file ijms-21-09637-s001.pdf]

SUPPLEMENTARY DATA

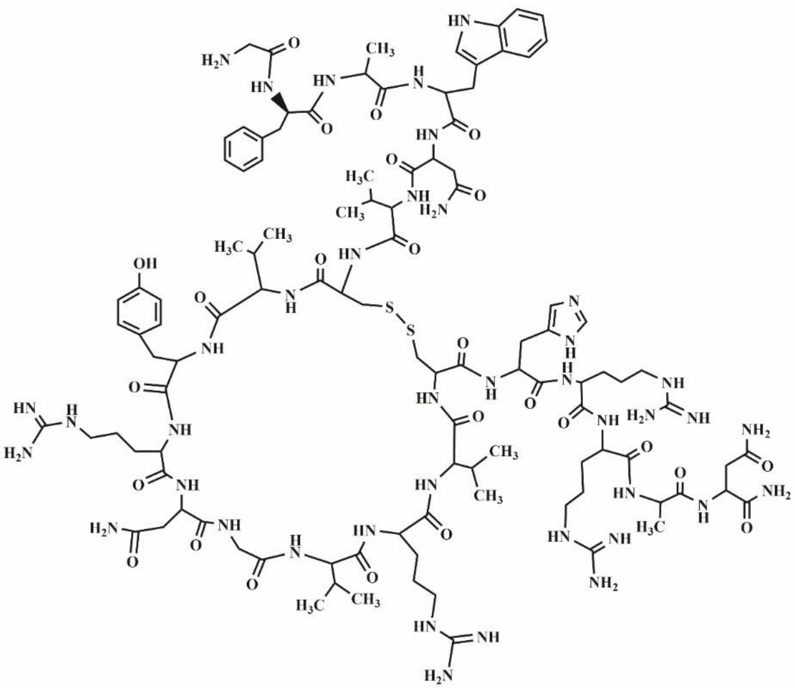

Figure S1-1. The chemical structure of $N6NH_2$.

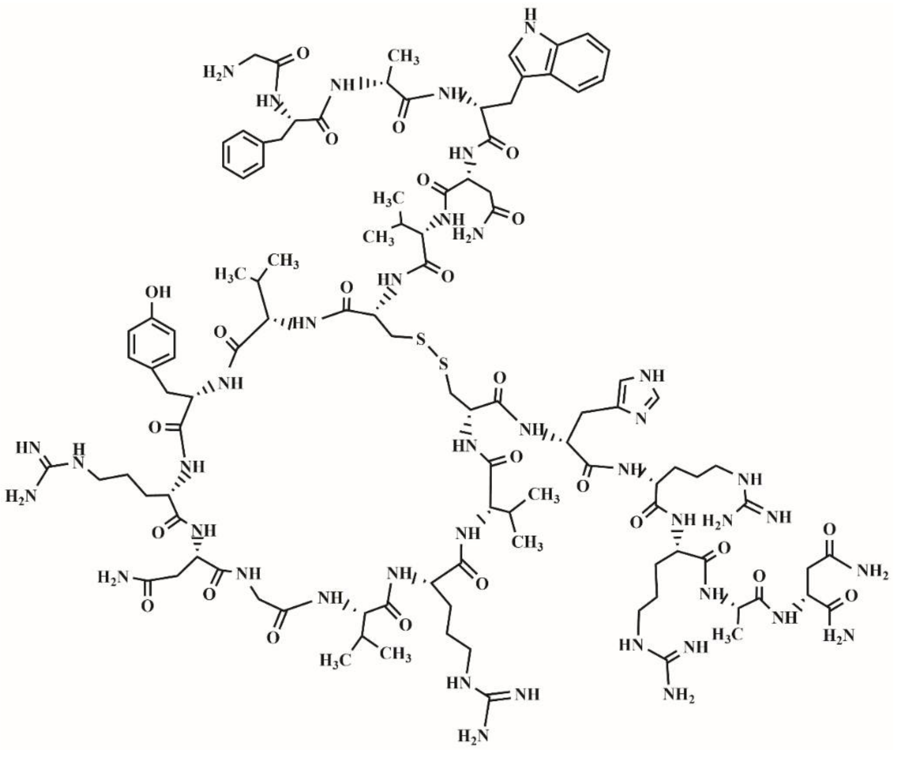

Figure S1-2. The chemical structure of $DN6NH_2$.

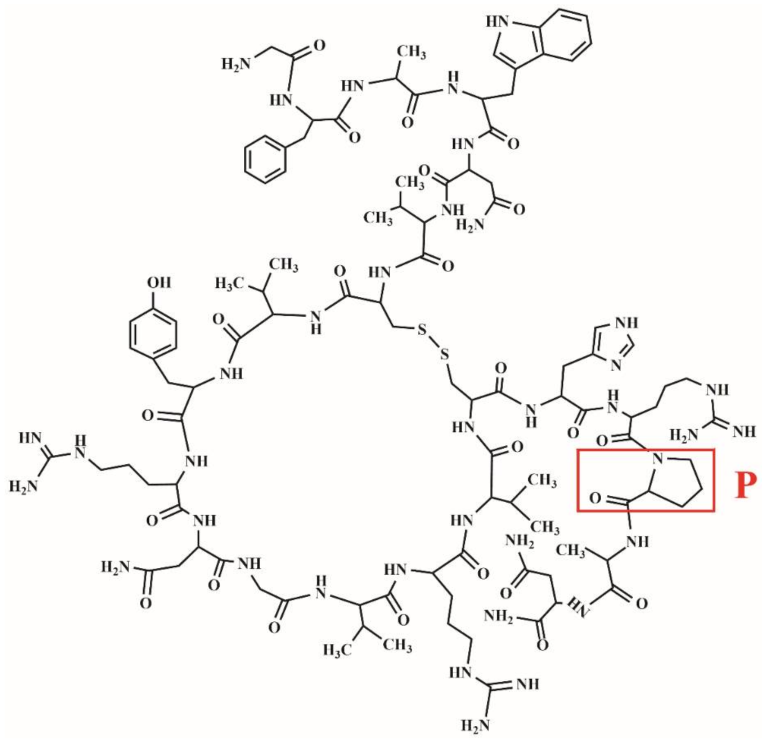

Figure S1-3. The chemical structure of N6PNH2. The red circle represents the position of amino acid replacement group.

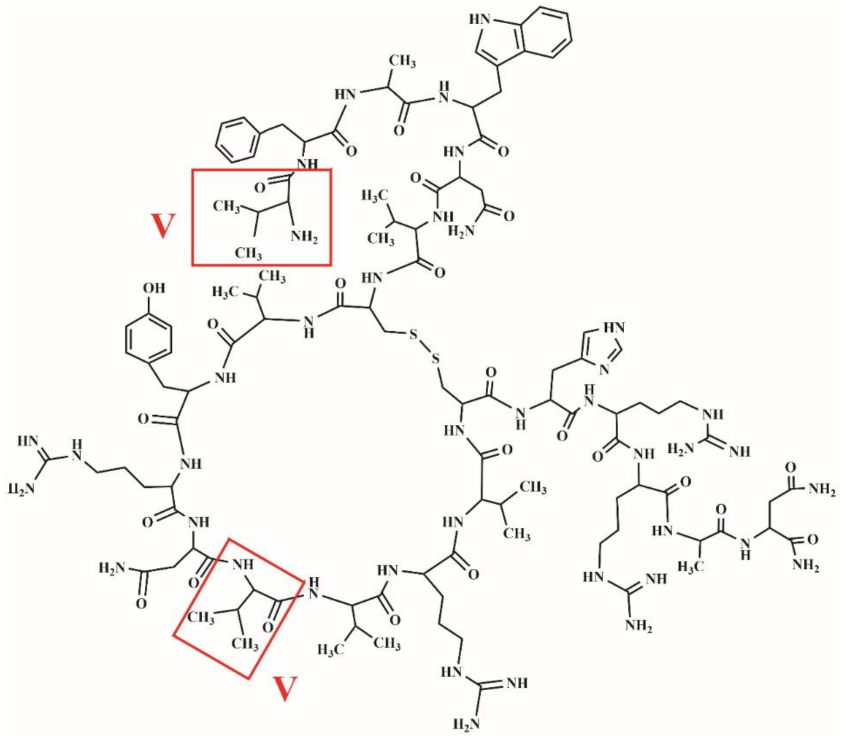

Figure S1-4. The chemical structure of V112N6NH2. The red circle represents the position of amino acid replacement group.

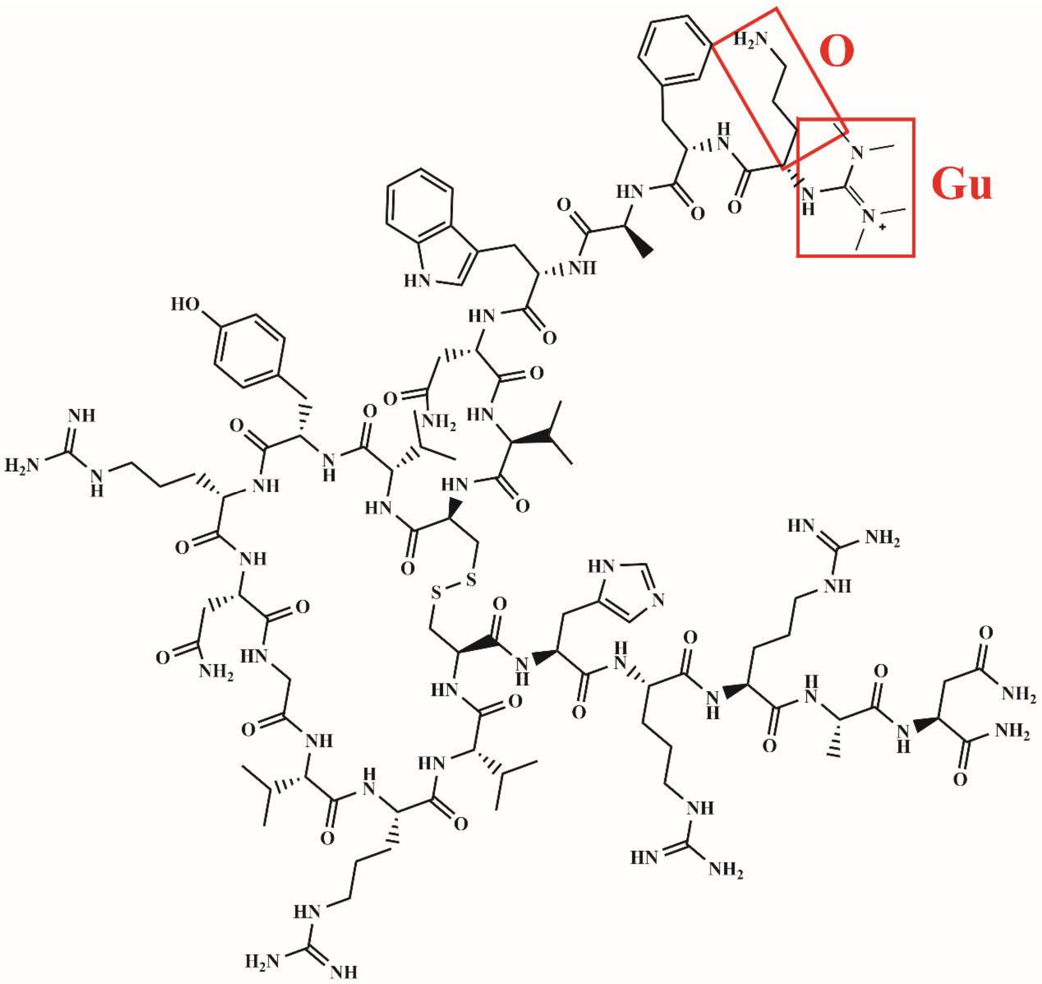

Figure S1-5. The chemical structure of Guo-N6NH_2. The red circle represents the position of amino acid added group.

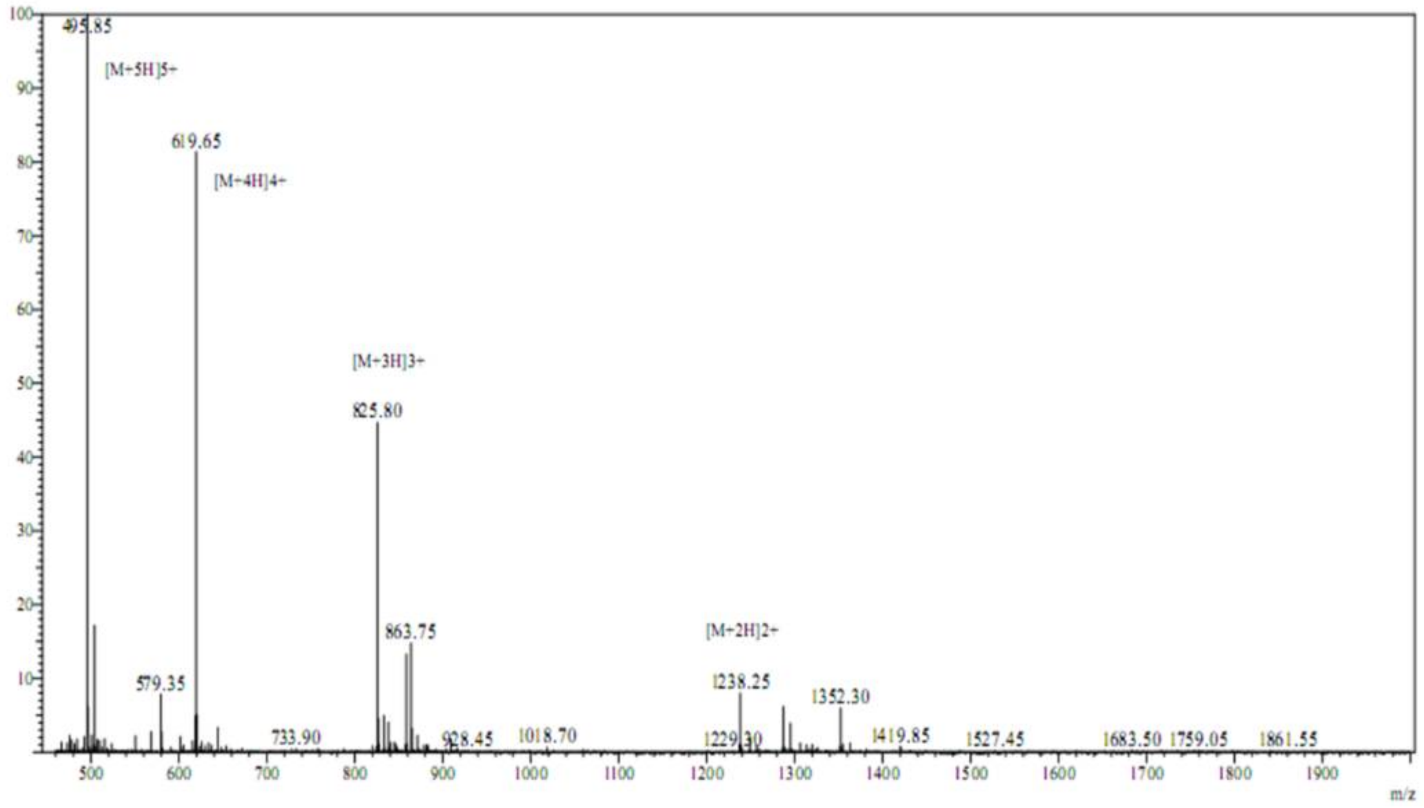

Figure S2-1. The mass spectrometry (MS) analysis of N6NH2.

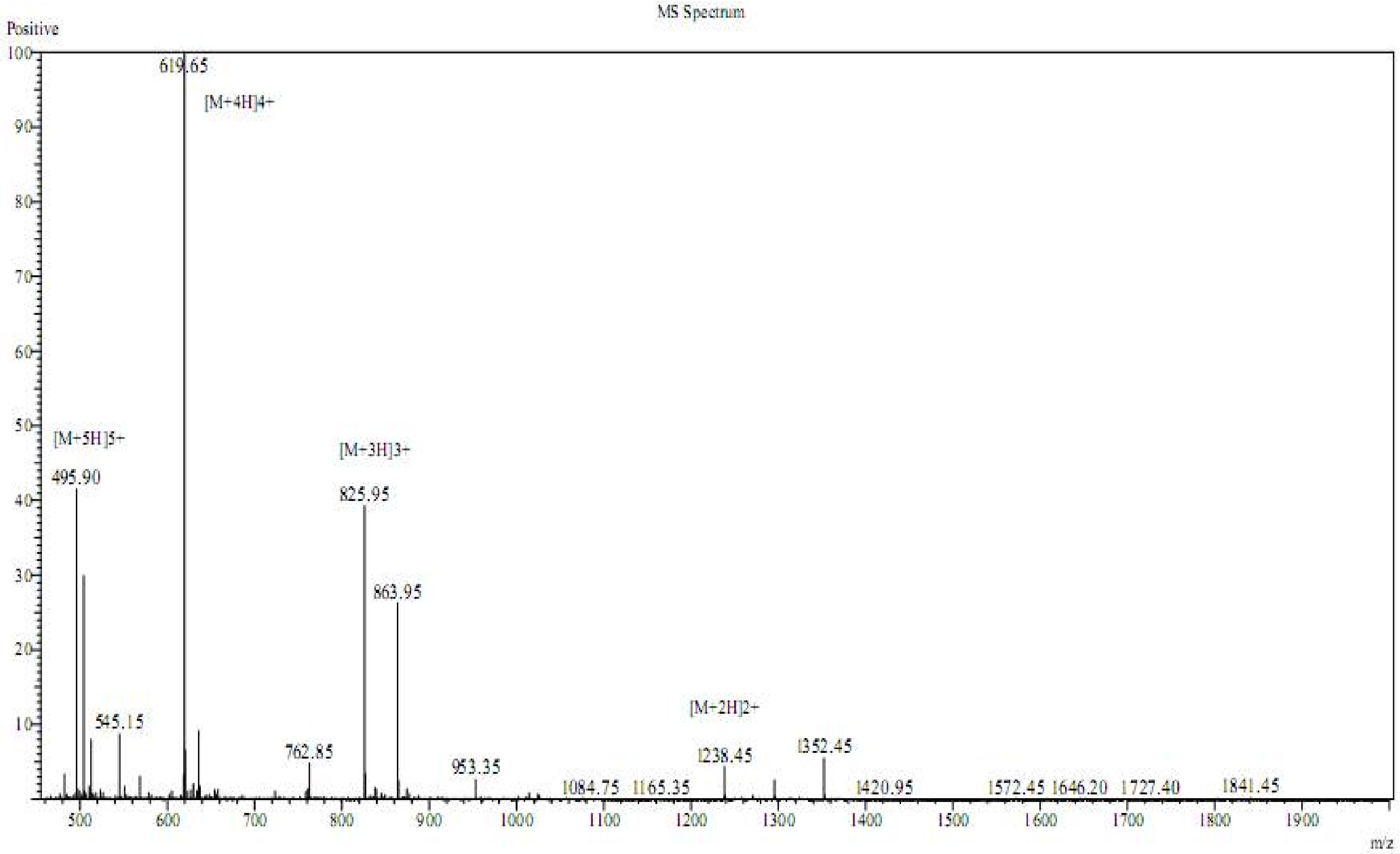

Figure S2-2. The mass spectrometry (MS) analysis of DN6NH2.

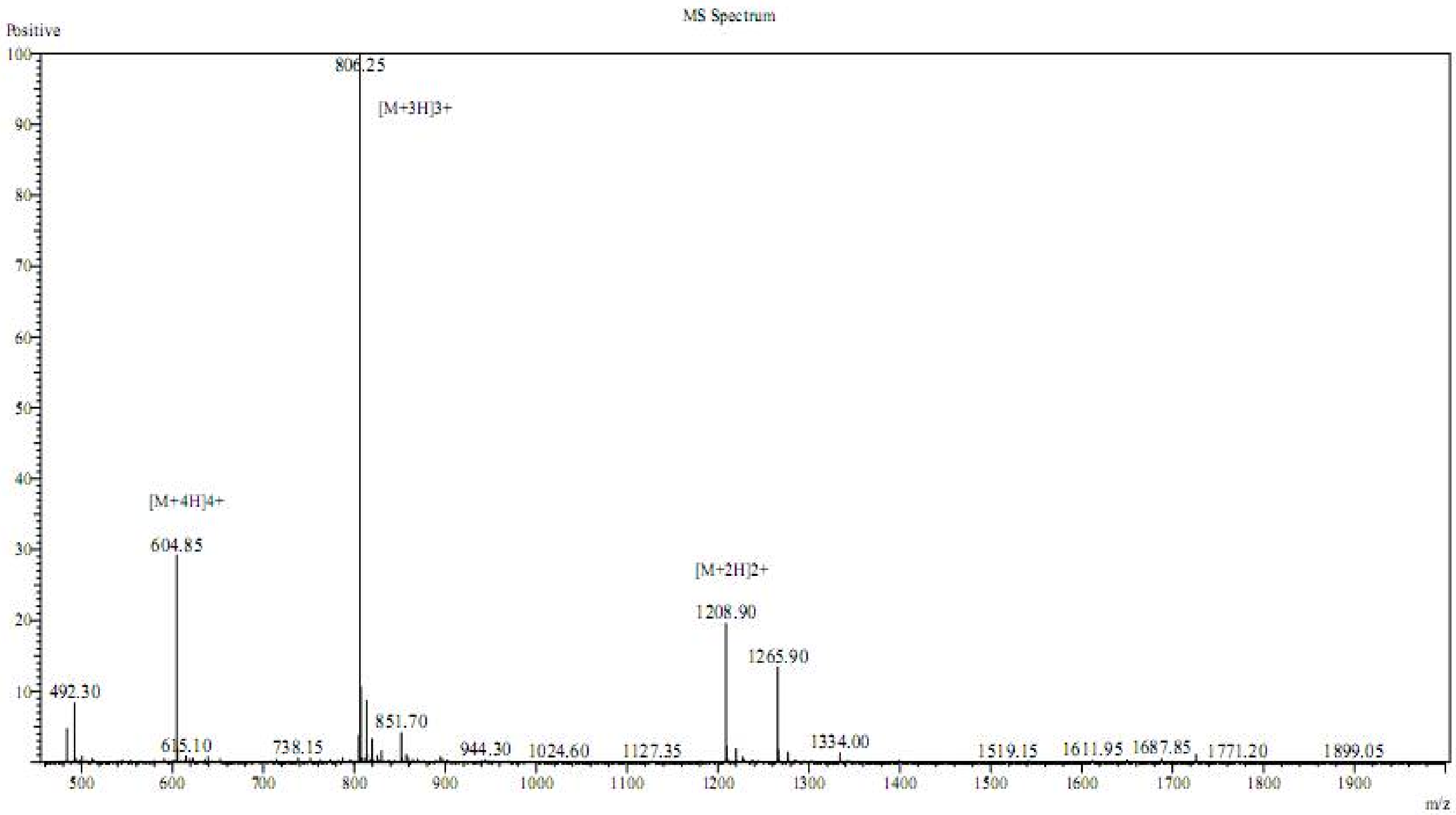

Figure S2-3. The mass spectrometry (MS) analysis of $N6PNH_2$.

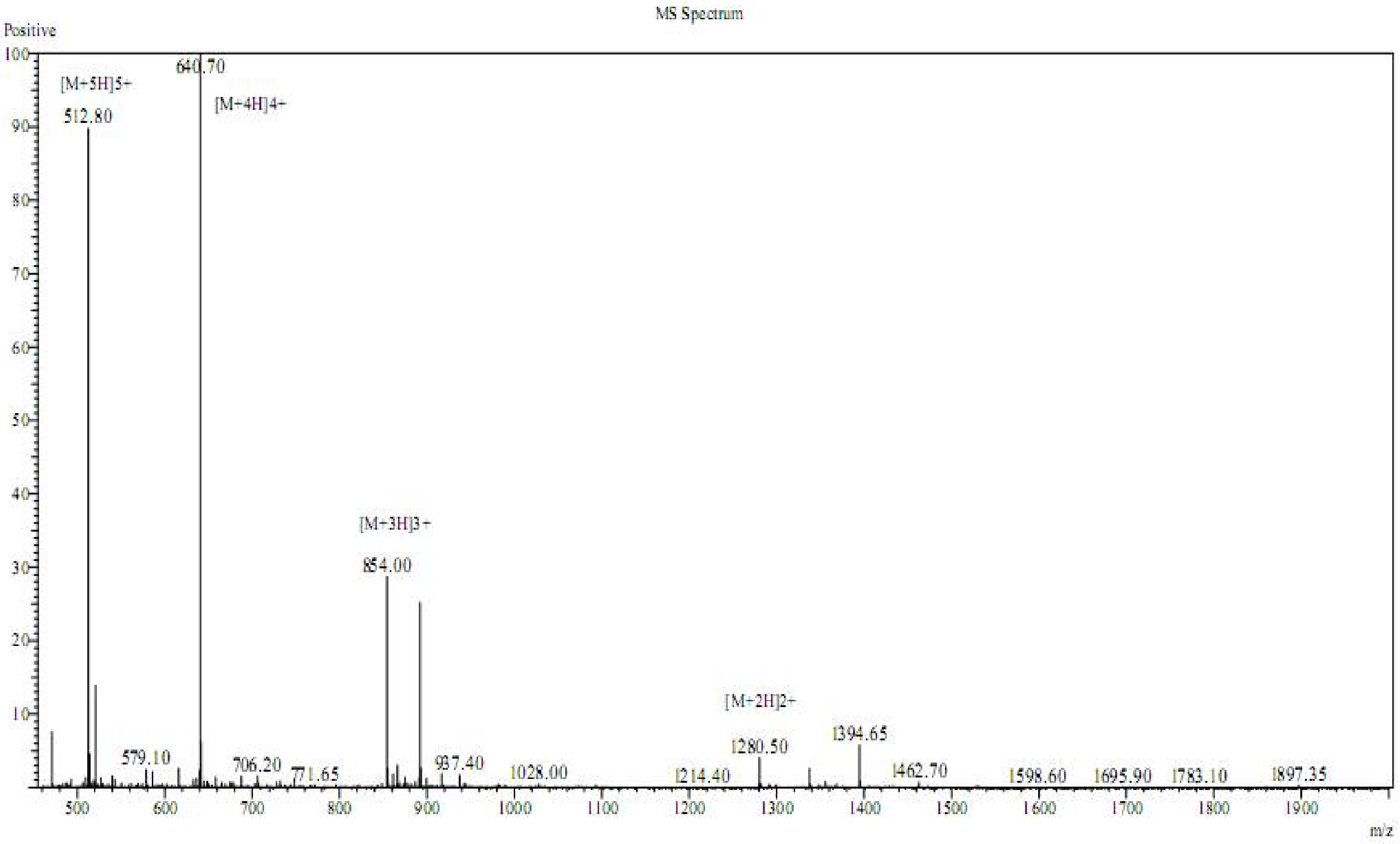

Figure S2-4. The mass spectrometry (MS) analysis of V112N6NH2.

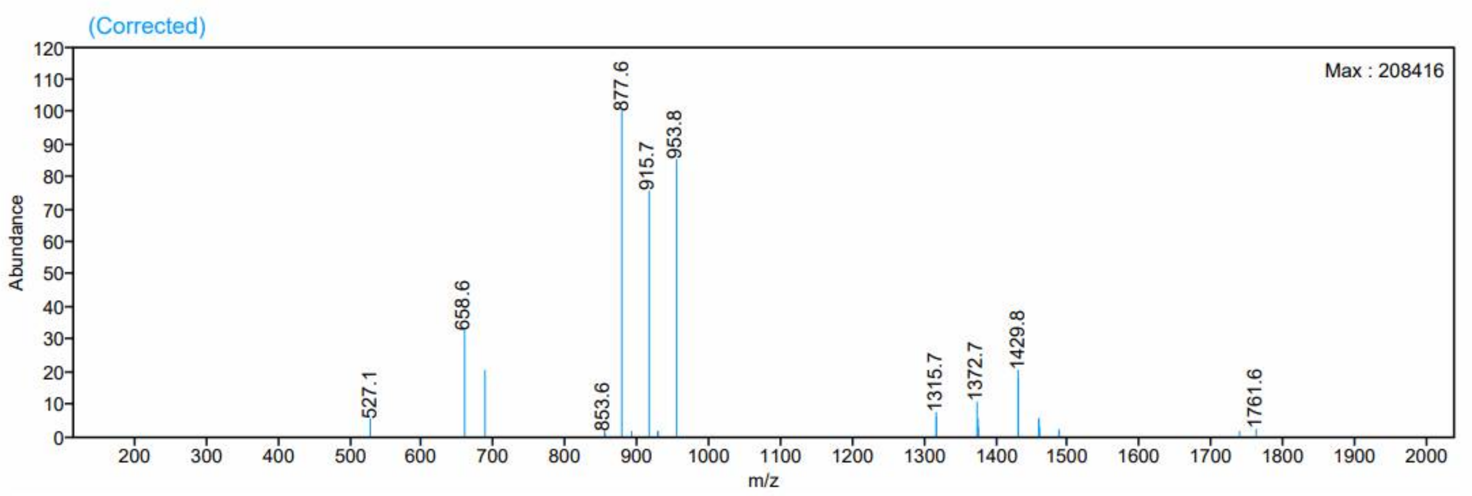

Figure S2-5. The mass spectrometry (MS) analysis of Guo-N6NH$_2$.

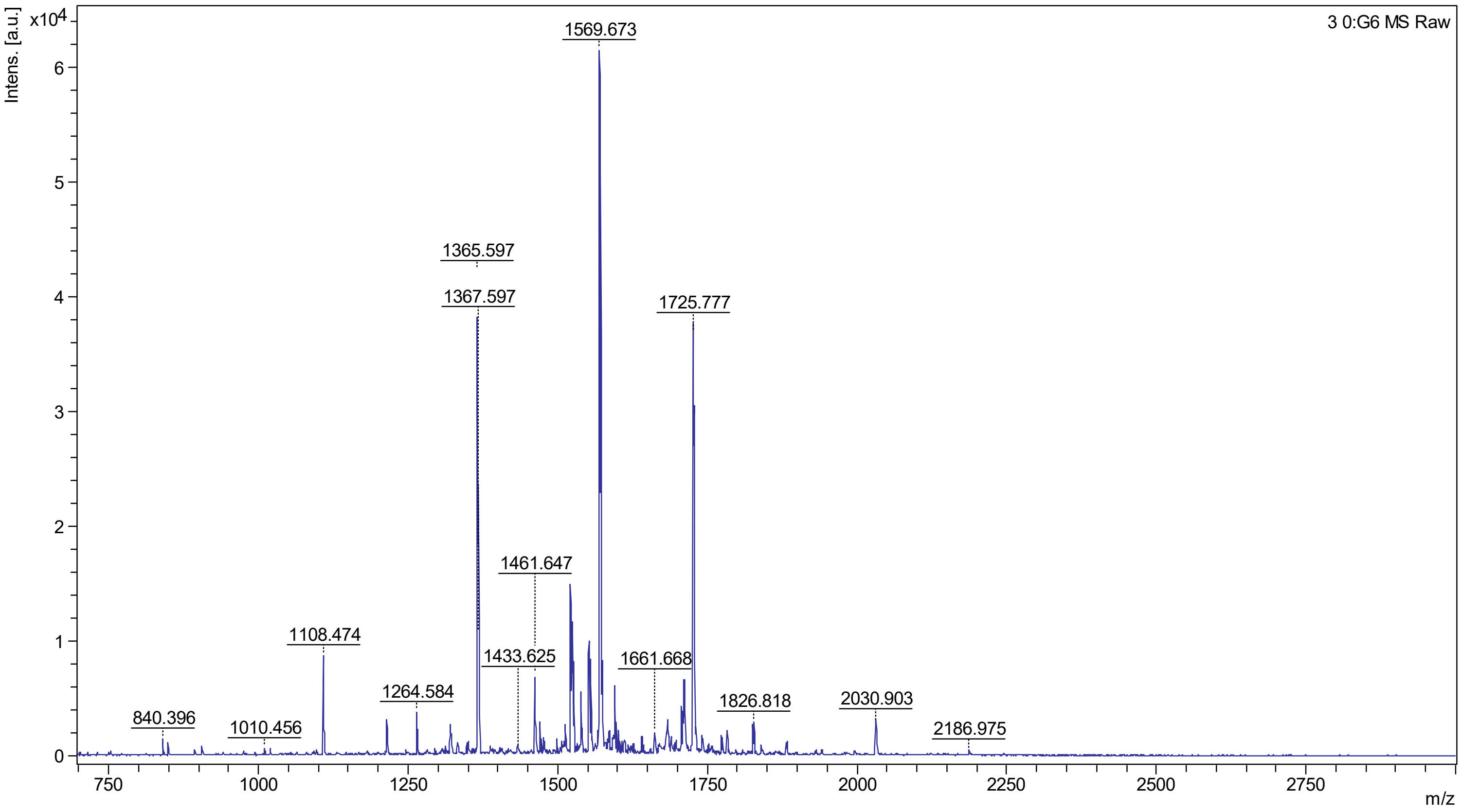

Figure S3. The mass spectrometry (MS) analysis of N6NH2 treated by trypsin. It was confirmed by MALDI-TOF MS (Ultraflextreme, Brucker, Germany) at the Laboratory of Proteomics, Institute of Biophysics, Chinese Academy of Sciences.

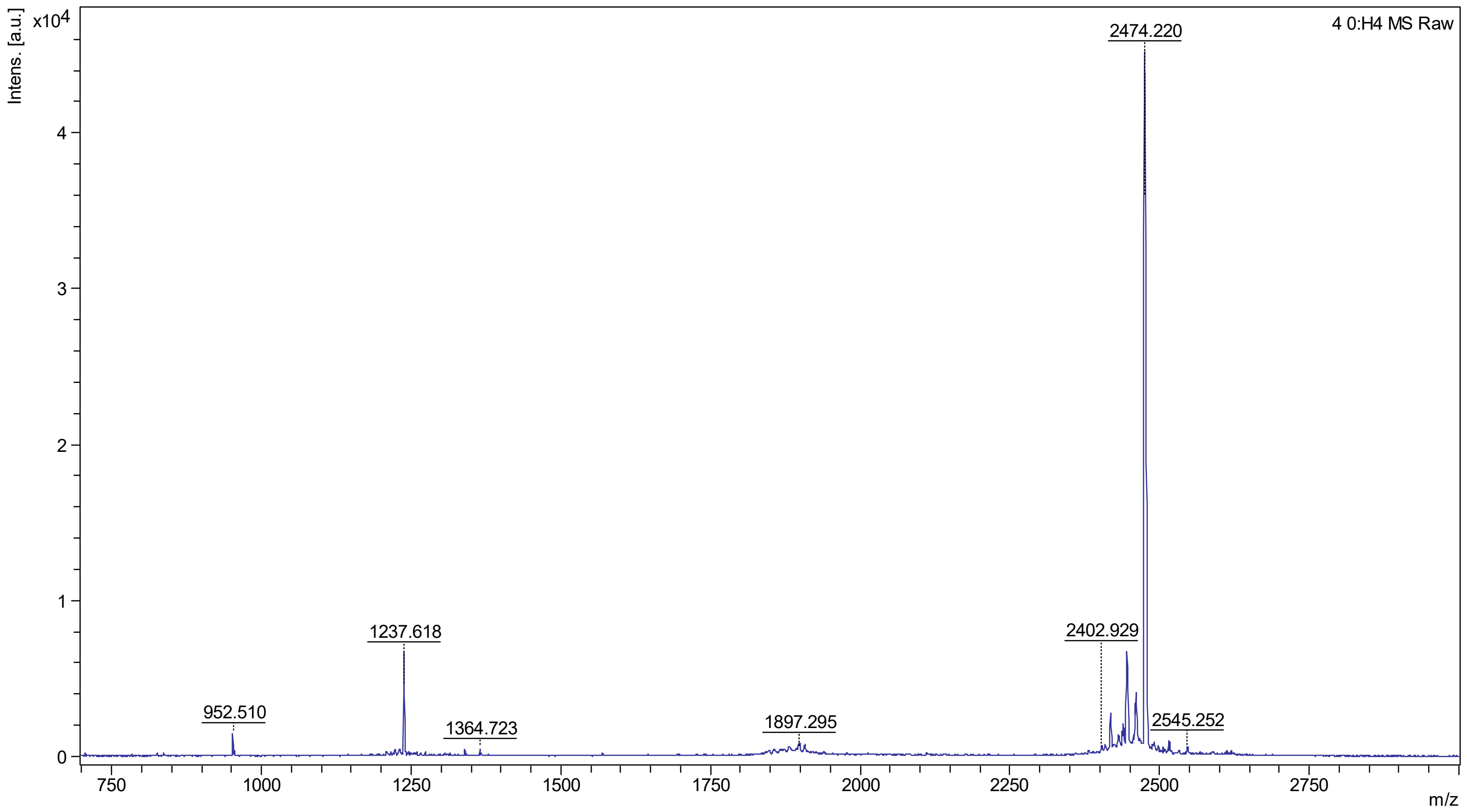

Figure S4. The MS analysis of $DN6NH_2$ treated by trypsin. It was confirmed by MALDI-TOF MS (Ultraflextreme, Brucker, Germany) at the Laboratory of Proteomics, Institute of Biophysics, Chinese Academy of Sciences.

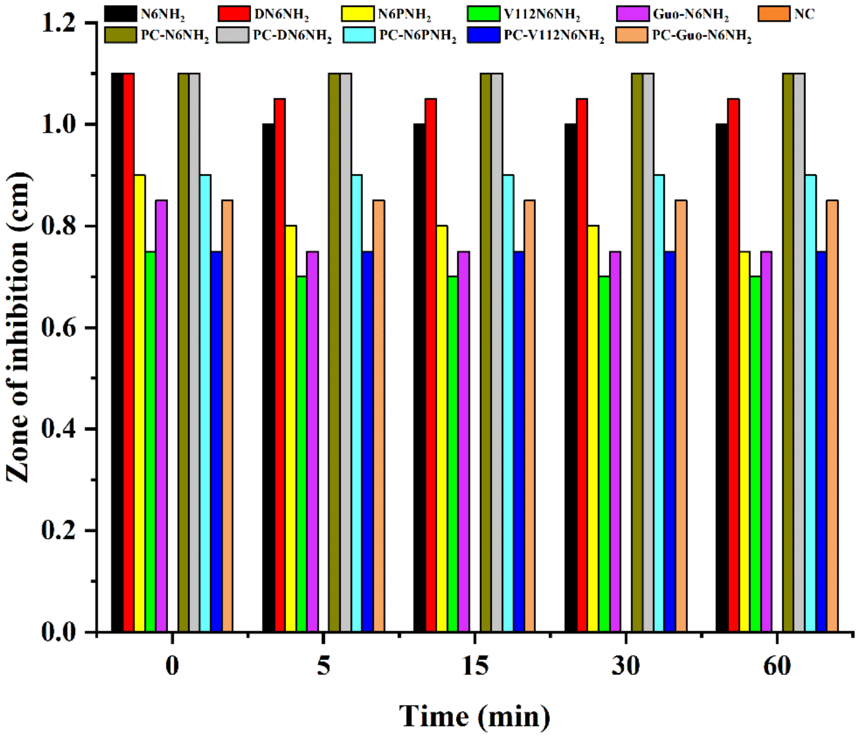

Figure S5. The stability of N6NH2 analogues in simulated gastric fluid (SGF). NC, negative control; PC, positive control.

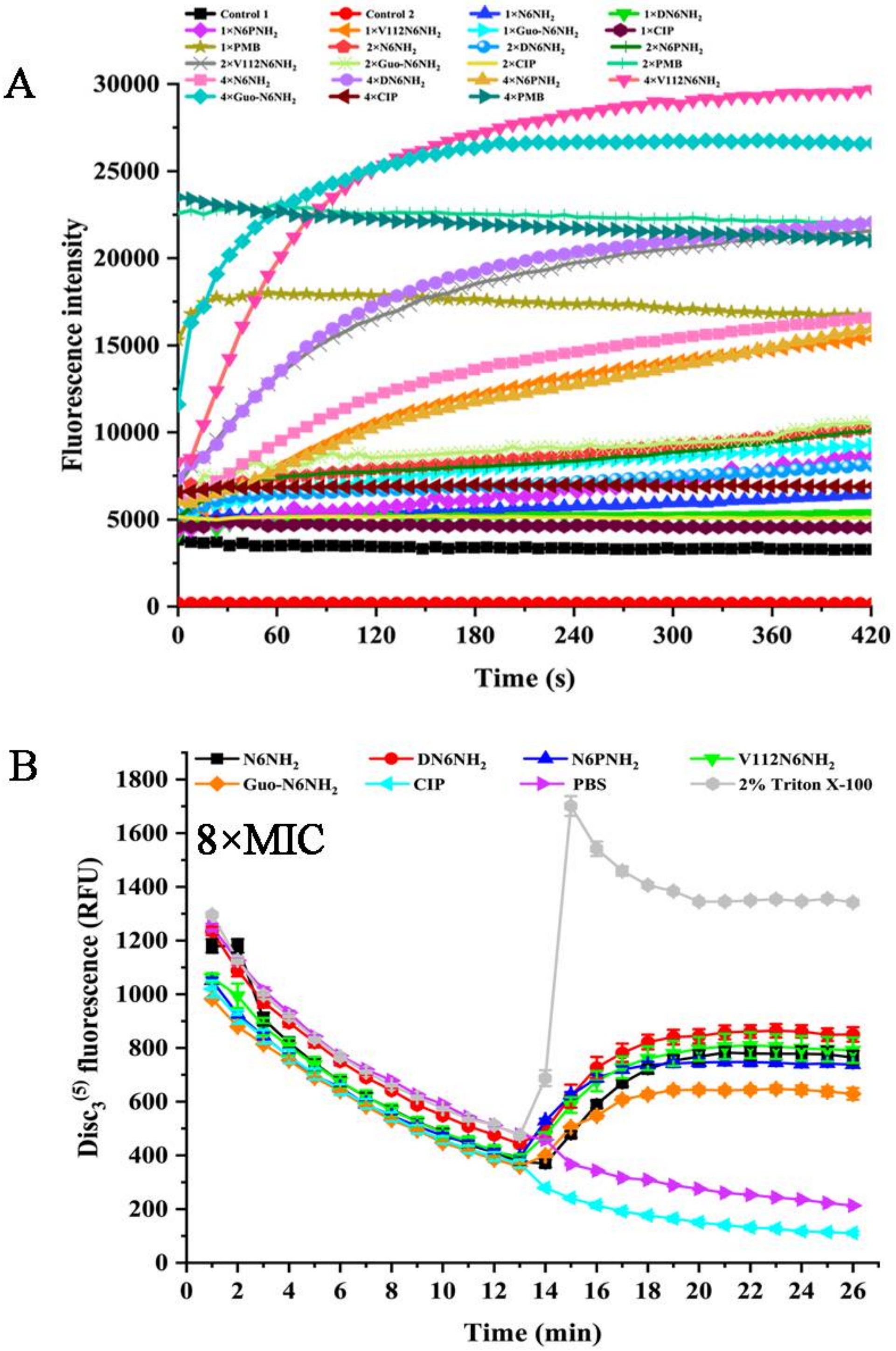

Figure S6-1. Interaction of $N6NH_2$ and its analogues with cell membrane. (**A**) $N6NH_2$ and its analogues outer membrane penetration test. (**B**) Effects of 8×MIC $N6NH_2$ and its analogues on bacterial cytoplasmic membrane potential.

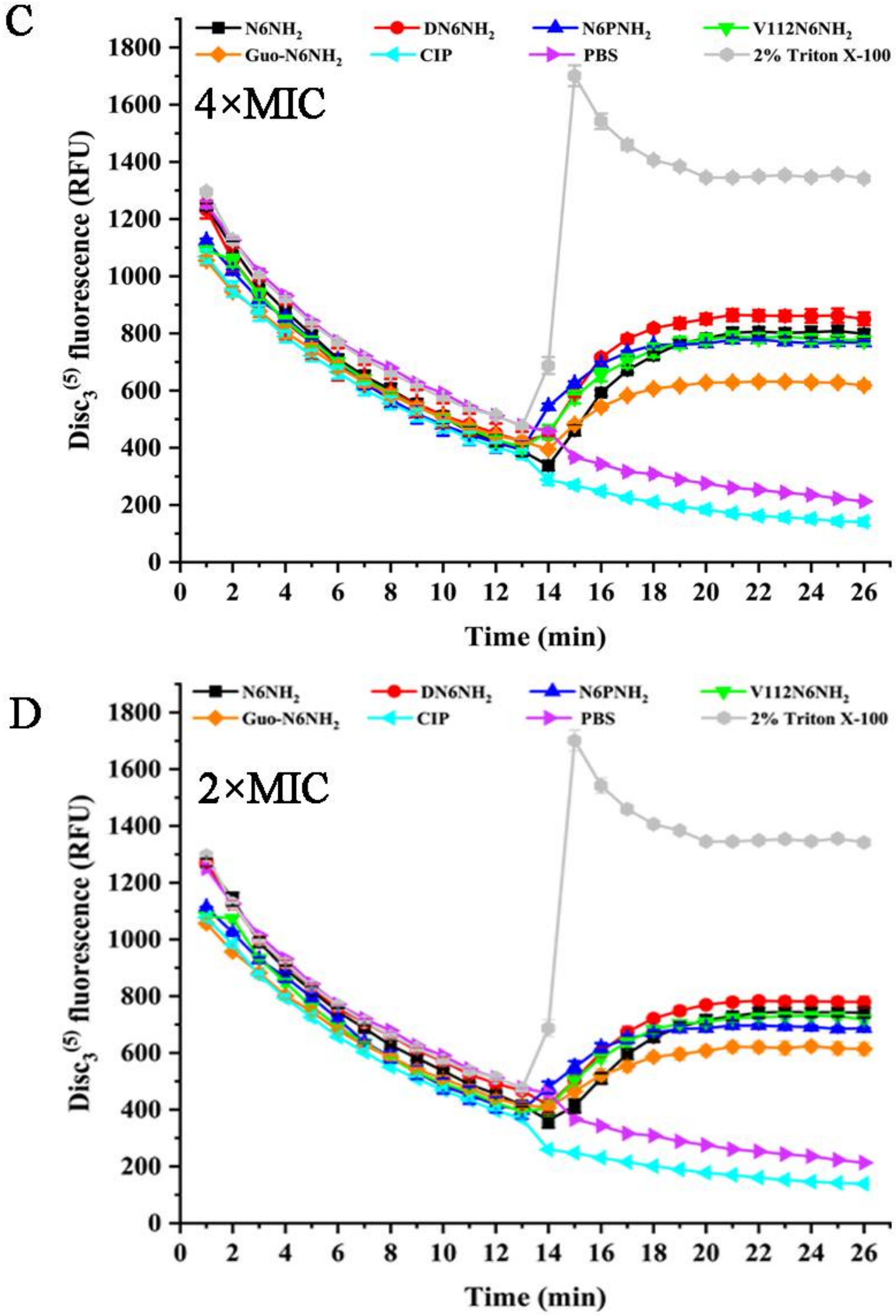

Figure S6-2. Interaction of N6NH2 and its analogues with cell membrane. (**C-D**) Effects of 4×MIC or 2×MIC N6NH2 and its analogues on bacterial cytoplasmic membrane potential.

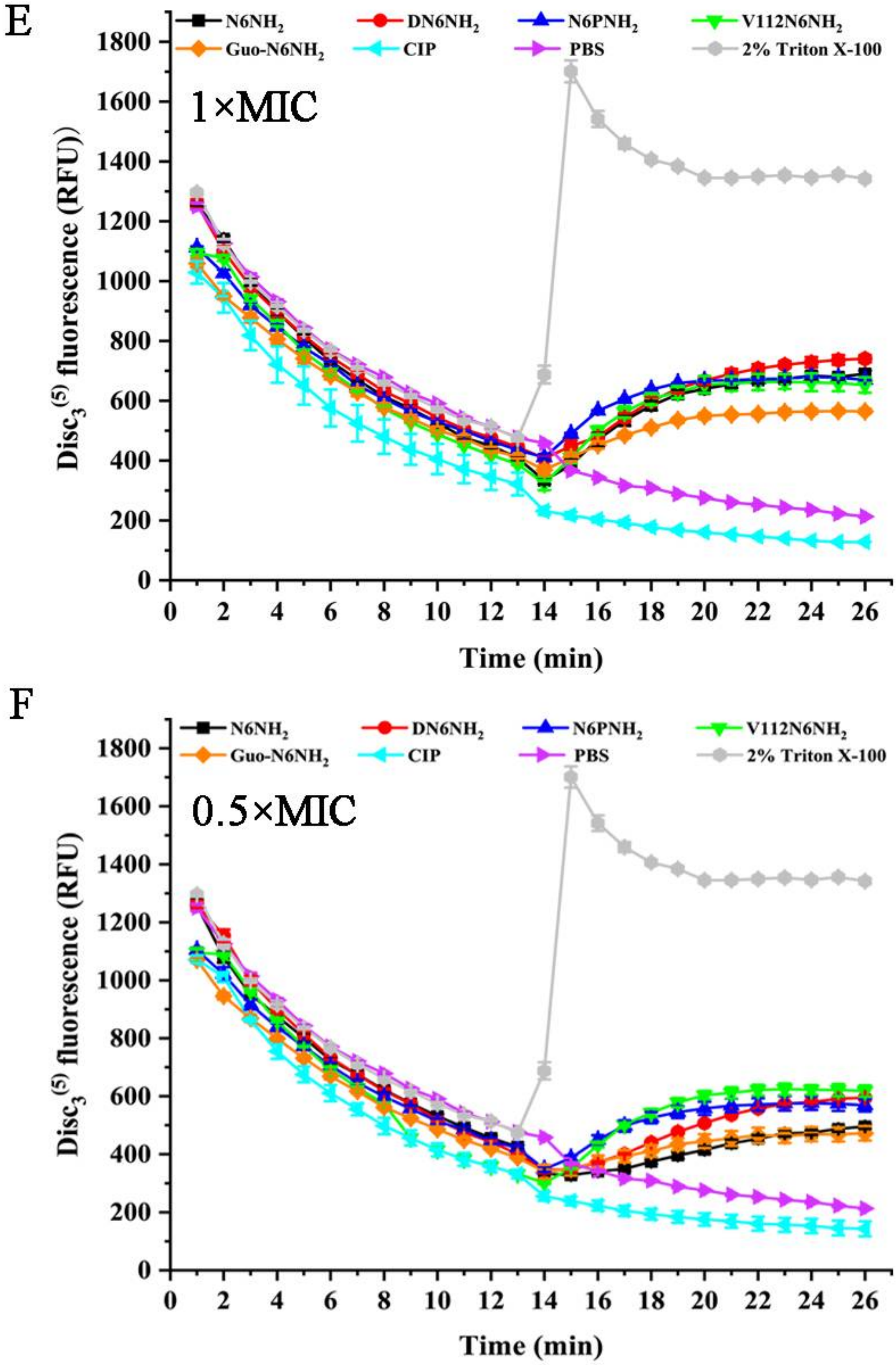

Figure S6-3. Interaction of N6NH2 and its analogues with cell membrane. (**E-F**) Effects of 1×MIC or 0.5×MIC N6NH2 and its analogues on bacterial cytoplasmic membrane potential.

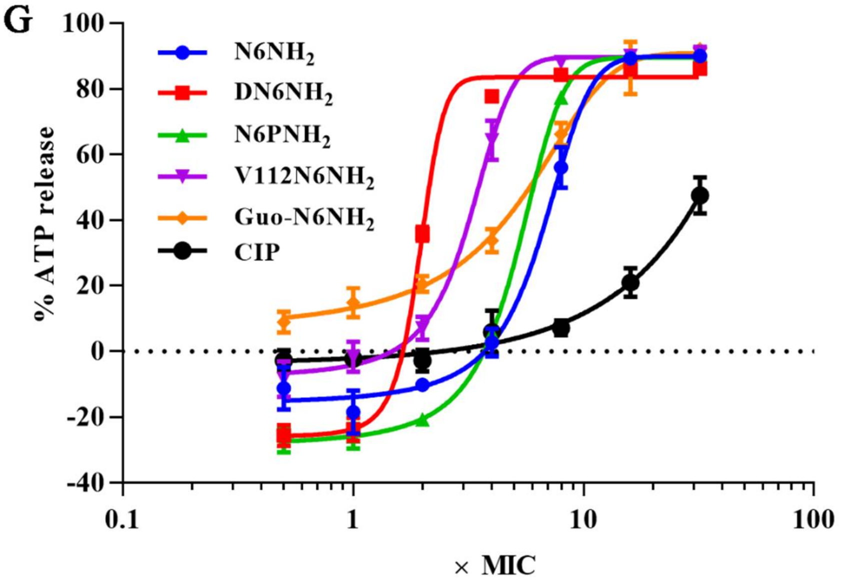

Figure S6-4. Interaction of N6NH2 and its analogues with cell membrane. (**G**) Extracellular ATP release.

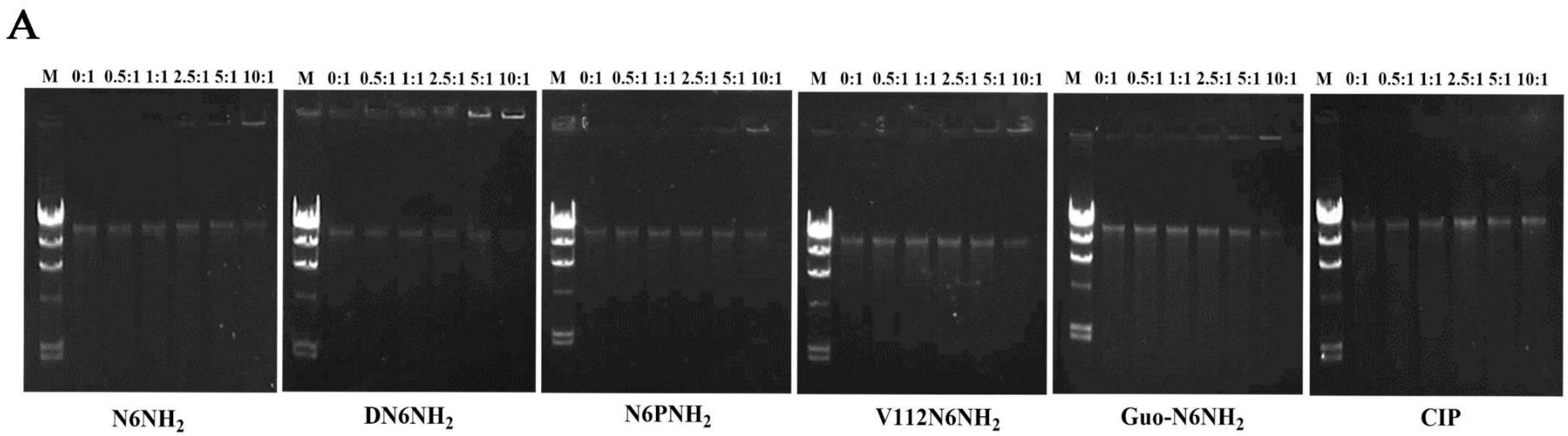

Figure S7-1. Interaction of $N6NH_2$ and its analogues with genomic DNA. (**A**) Gel block analysis of interaction between $N6NH_2$ analogues and genomic DNA of *A. veronii* ACCC61732.

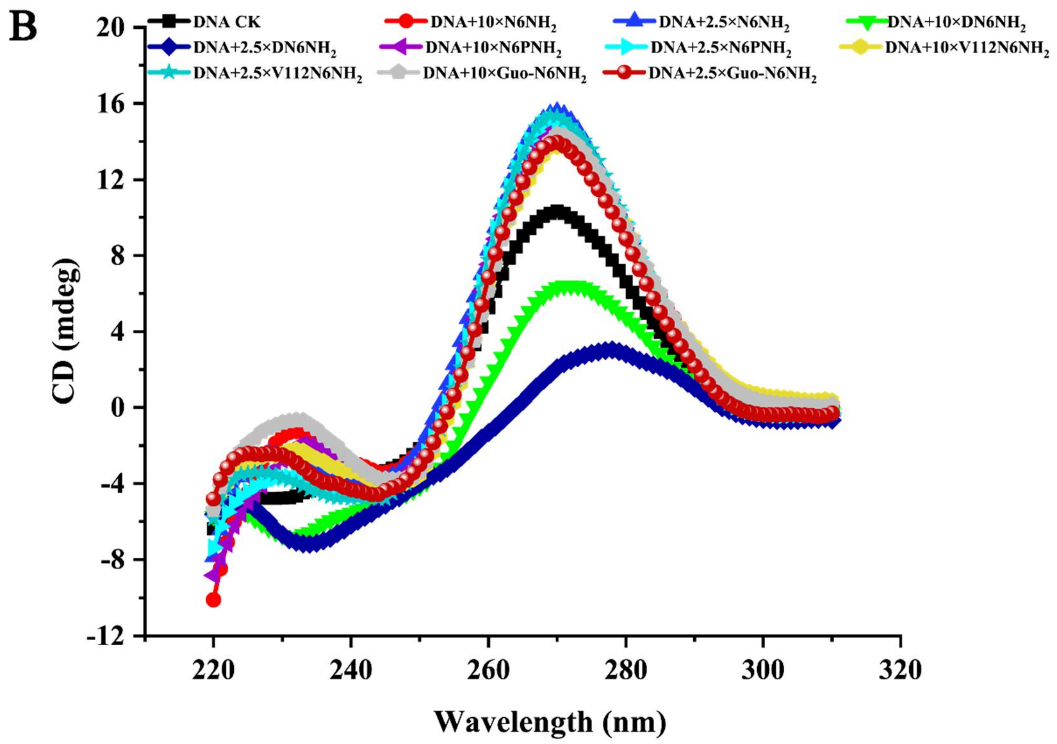

Figure S7-2. Interaction of N6NH2 and its analogues with genomic DNA. (**B**) CD analysis of the effect of N6NH2 and its analogues on DNA structure.

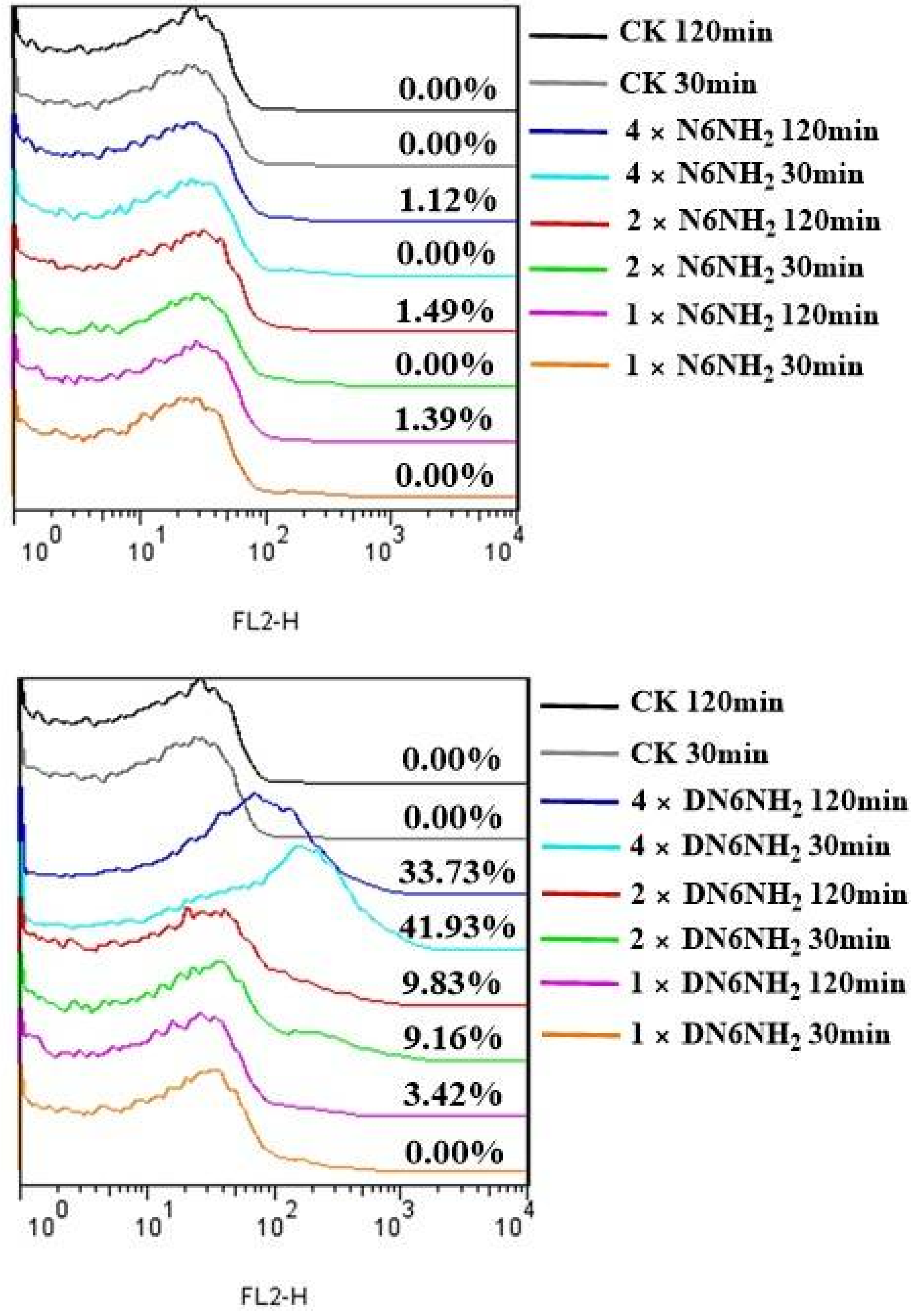

Figure S8-1. FACS analysis of the membrane penetration capacity of N6NH_2 and DN6NH_2 against *A. veronii* ACCC61732.

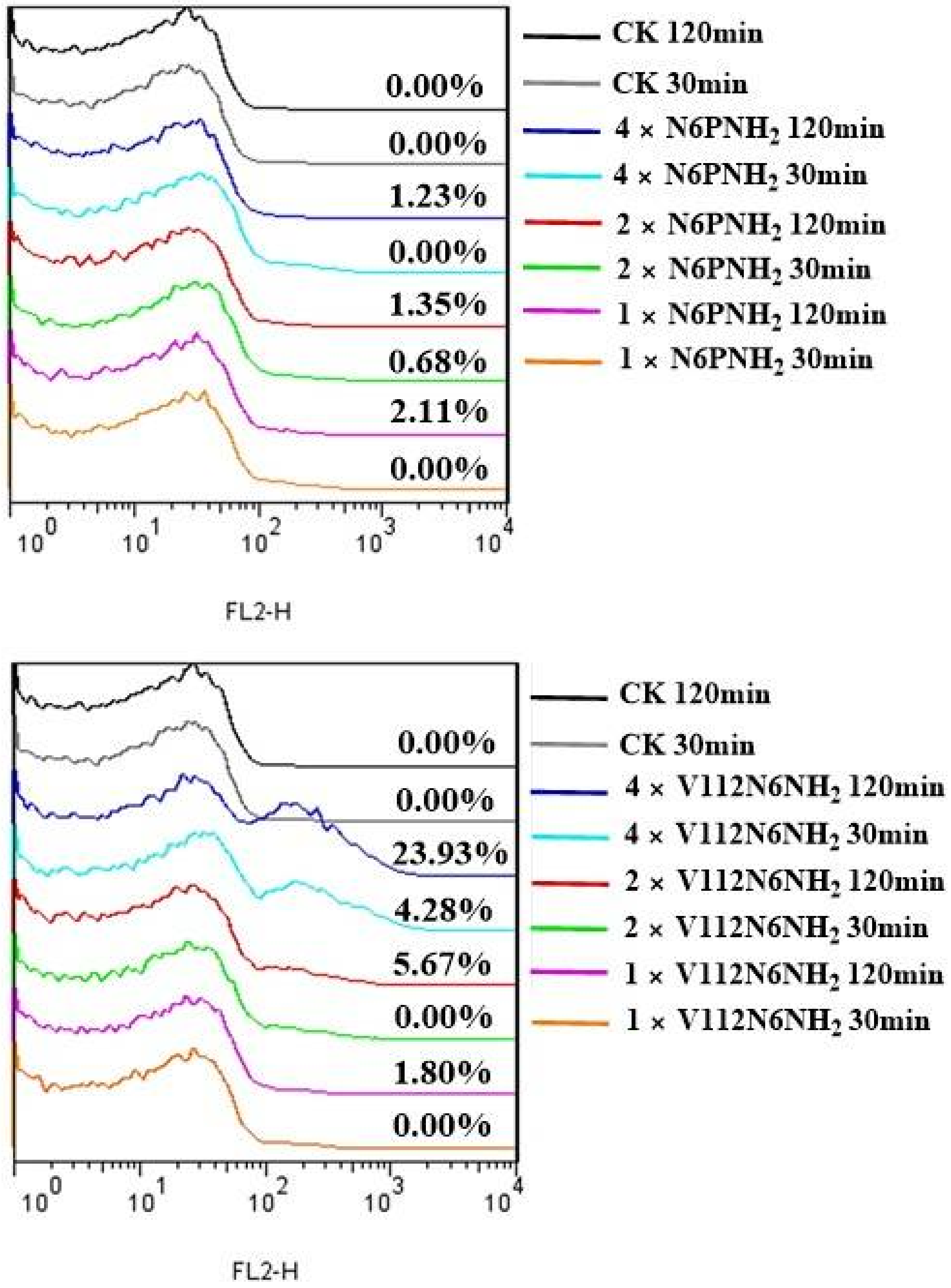

Figure S8-2. FACS analysis of the membrane penetration capacity of $N6PNH_2$ and $V112N6NH_2$ against *A. veronii* ACCC61732.

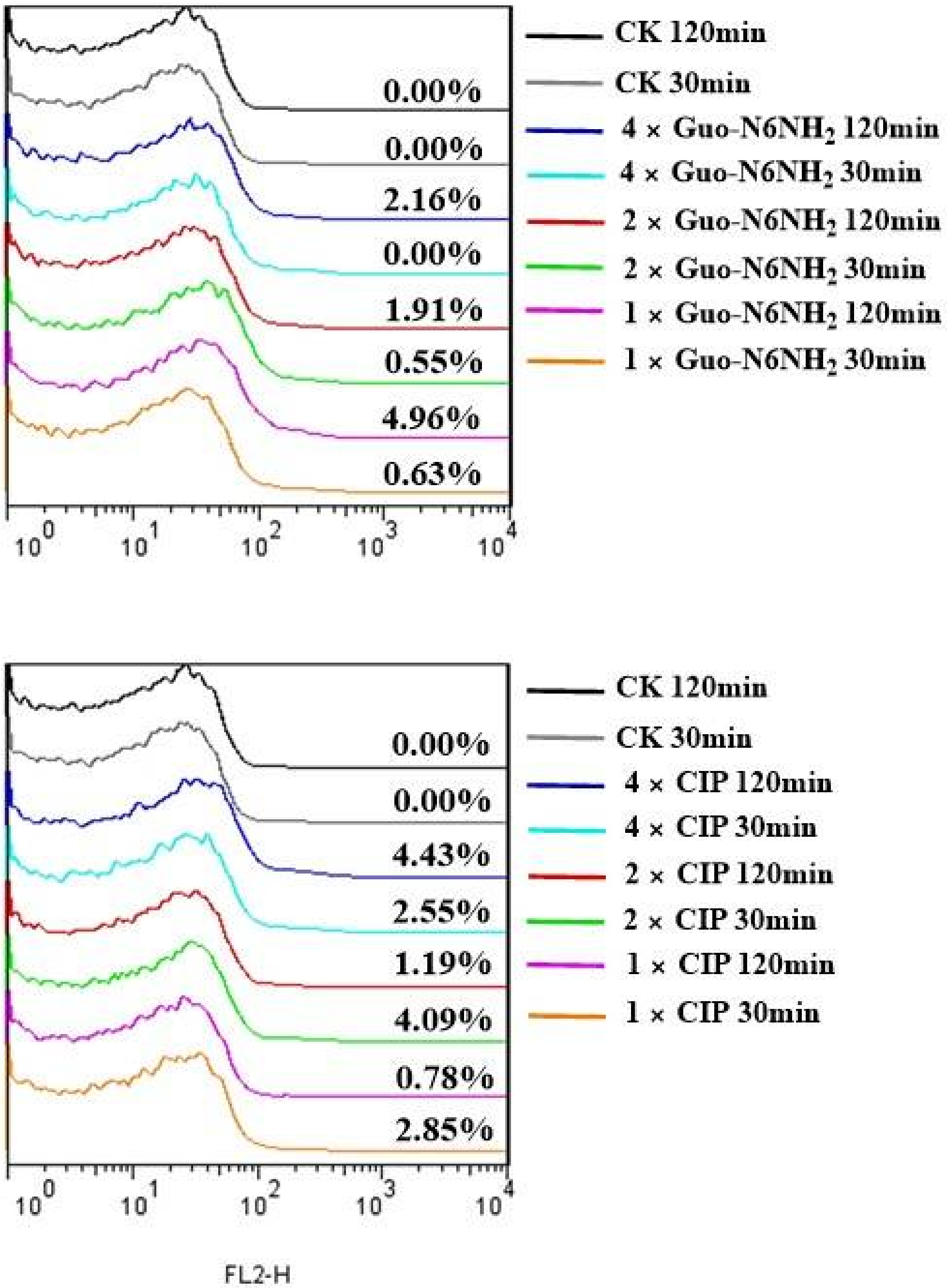

Figure S8-3. FACS analysis of the membrane penetration capacity of Guo-N6NH2 and CIP against *A. veronii* ACCC61732.

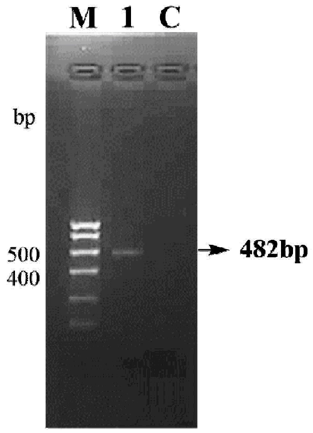

Figure S9. Identification of *vac* J gene of *A. veronii* ACCC61732. Lane M: *Trans* DNA Marker I. Lanes 1: *vac*J gene. Lane C: negative control.

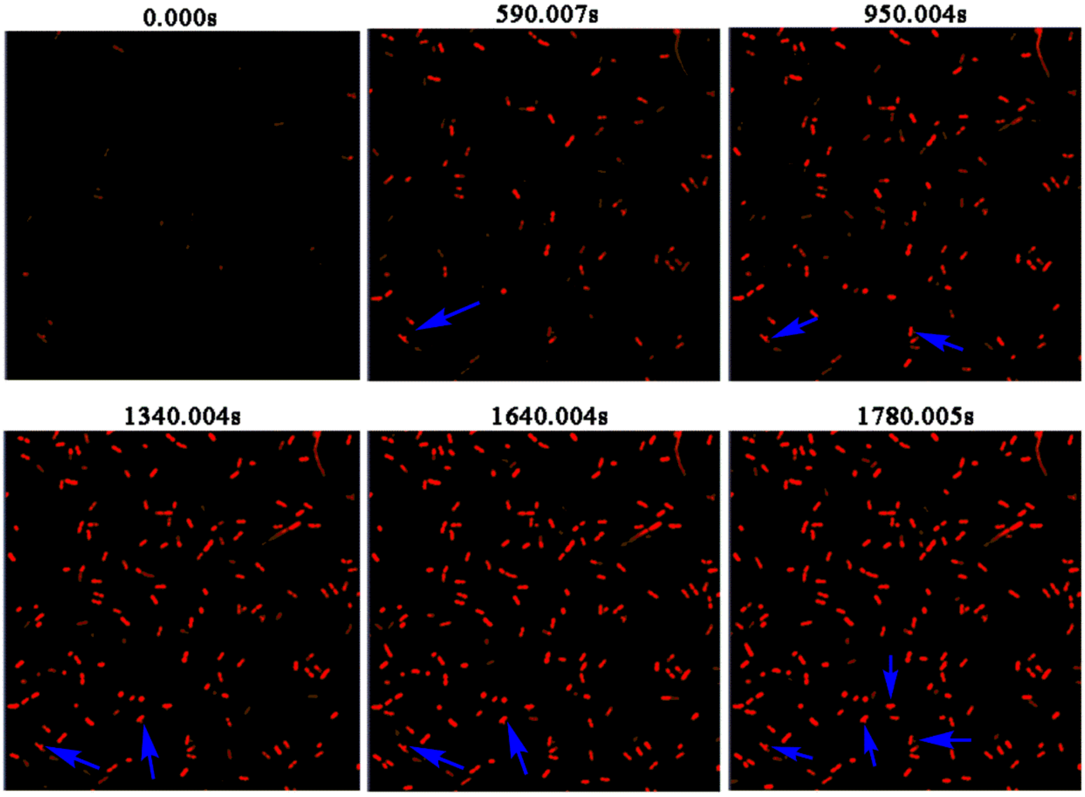

Figure S10. Outer membrane vesicles (OMVs) of *A. veronii* ACCC61732 treated with V112N6NH2. The cells were observed using CLSM.

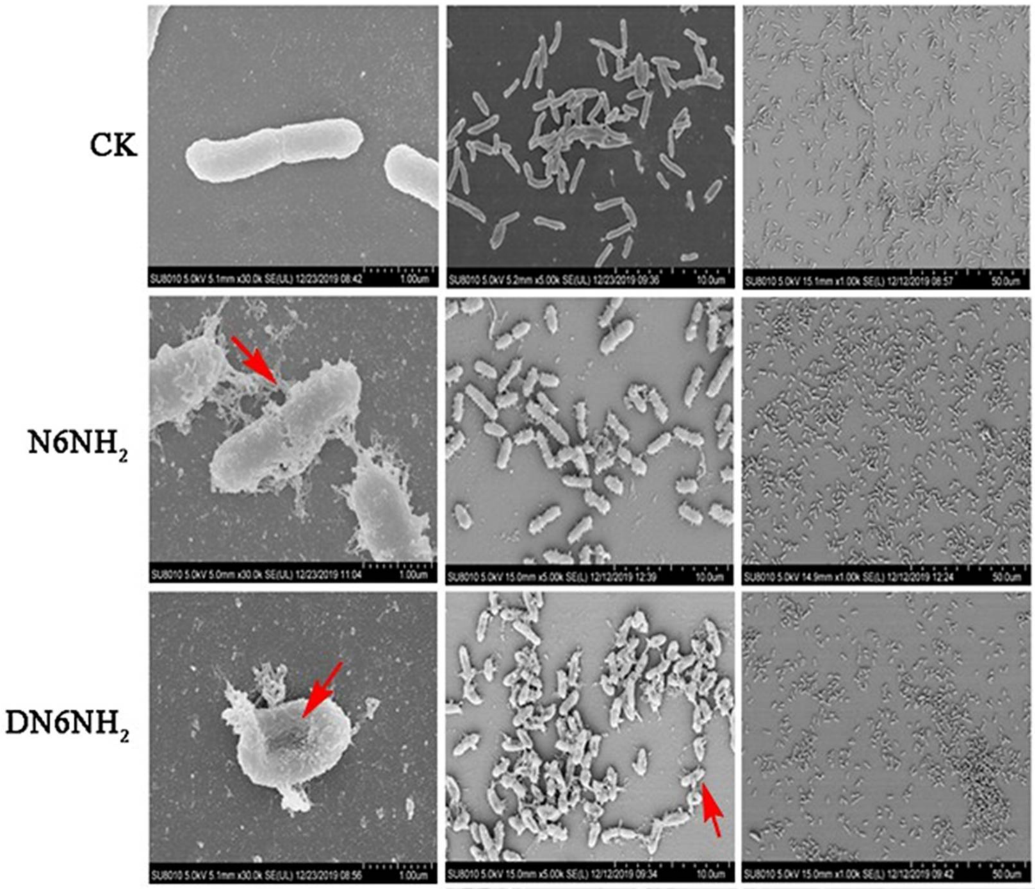

Figure S11-1. Effects of N6NH2 or DN6NH2 on biofilms of *A. veronii* ACCC61732 by using SEM.

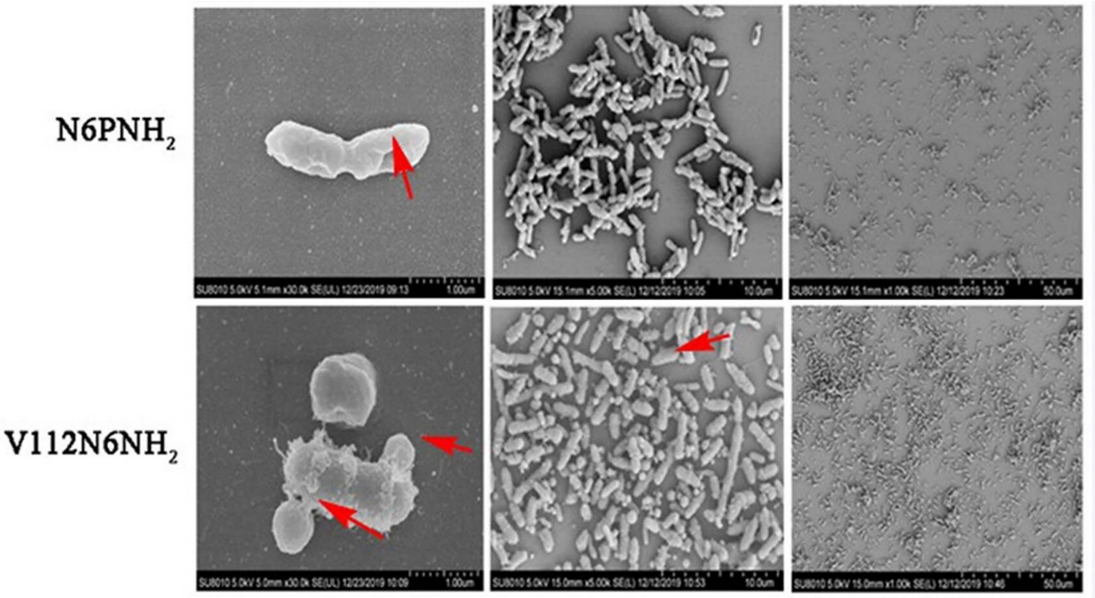

Figure S11-2. Effects of N6PNH2 or V112N6NH2 on biofilms of *A. veronii* ACCC61732 by using SEM.

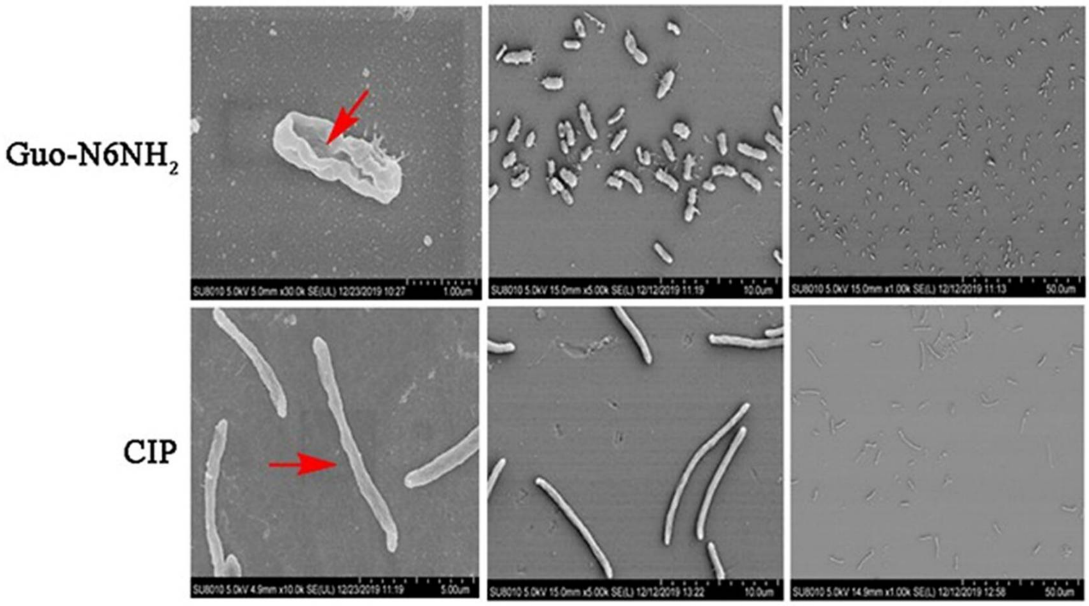

Figure S11-3. Effects of Guo-N6NH2 or CIP on biofilms of *A. veronii* ACCC61732 by using SEM.

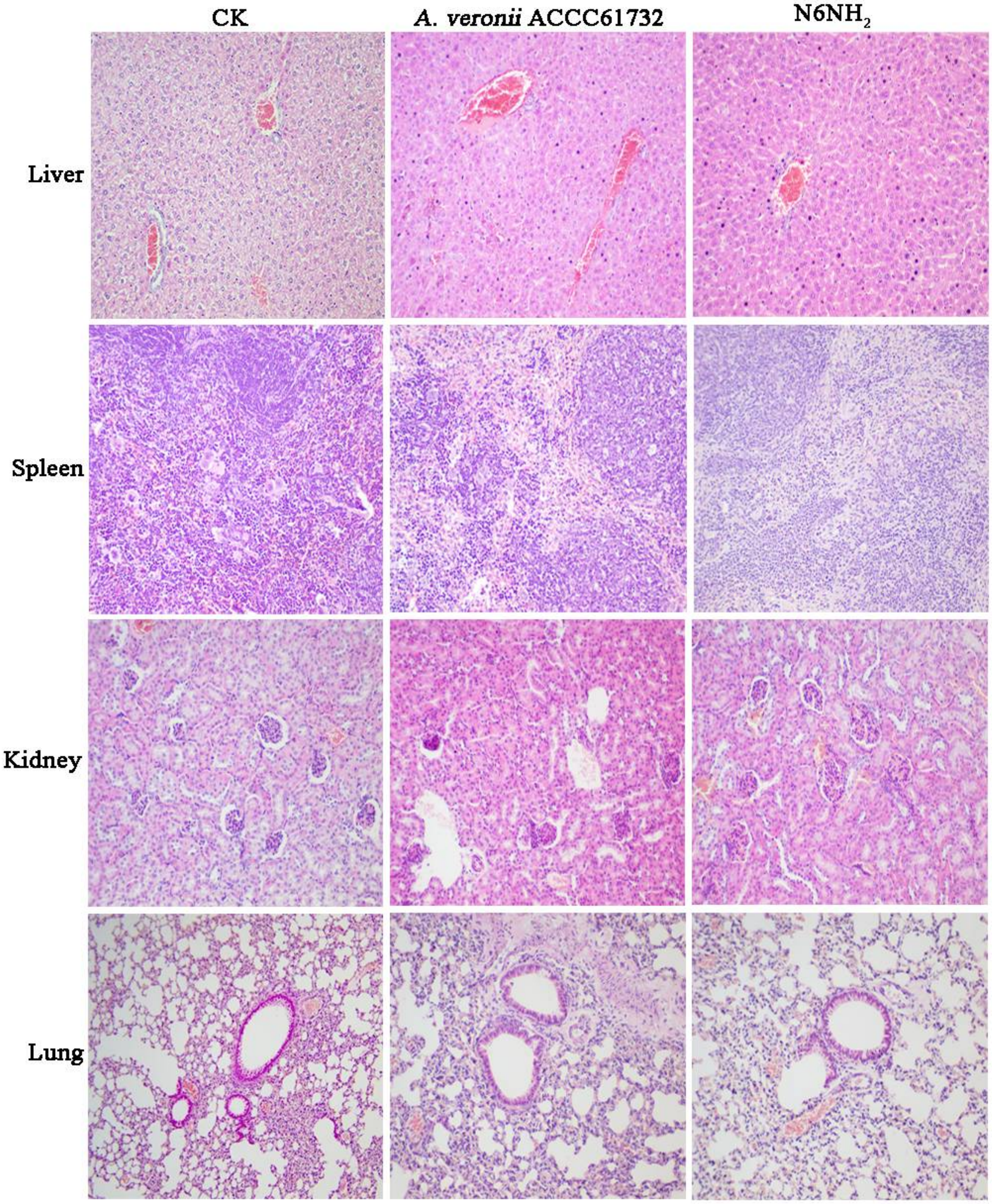

Figure S12-1. Effects of $N6NH_2$ and its analogues on organ injury in mice. Mice were infected intraperitoneally with *A. veronii* ACCC61732 (6×10^8 CFU/mL, 200 μL) and treated with $N6NH_2$ (5 μmol/kg). The livers, spleens, kidneys and lungs were harvested from mice sacrificed at 24 h after infection.

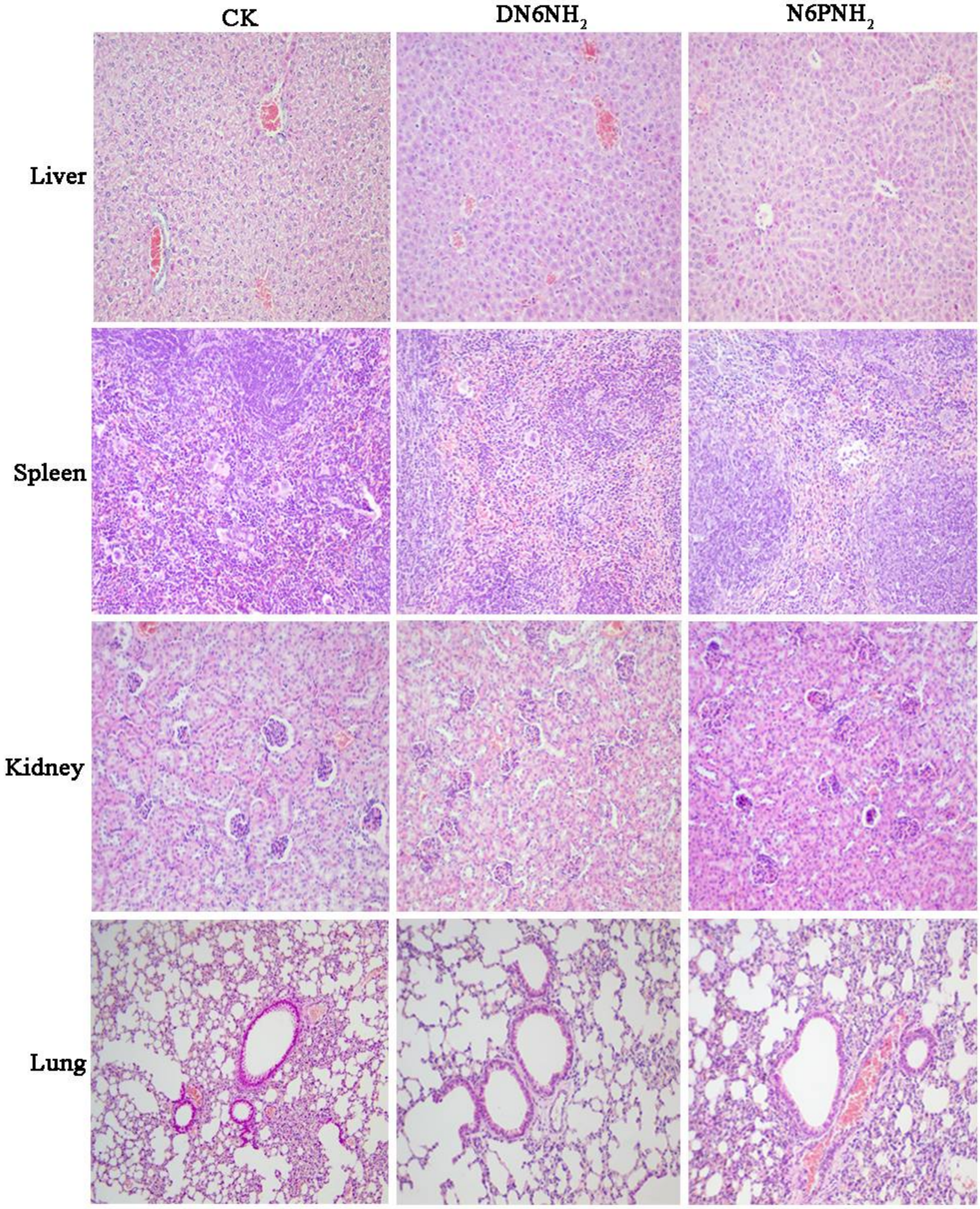

Figure S12-2. Effects of N6NH2 and its analogues on organ injury in mice. Mice were infected intraperitoneally with *A. veronii* ACCC61732 (6×10^8 CFU/mL, 200 μL) and treated with DN6NH2 and N6PNH2 (5 μmol/kg). The livers, spleens, kidneys and lungs were harvested from mice sacrificed at 24 h after infection.

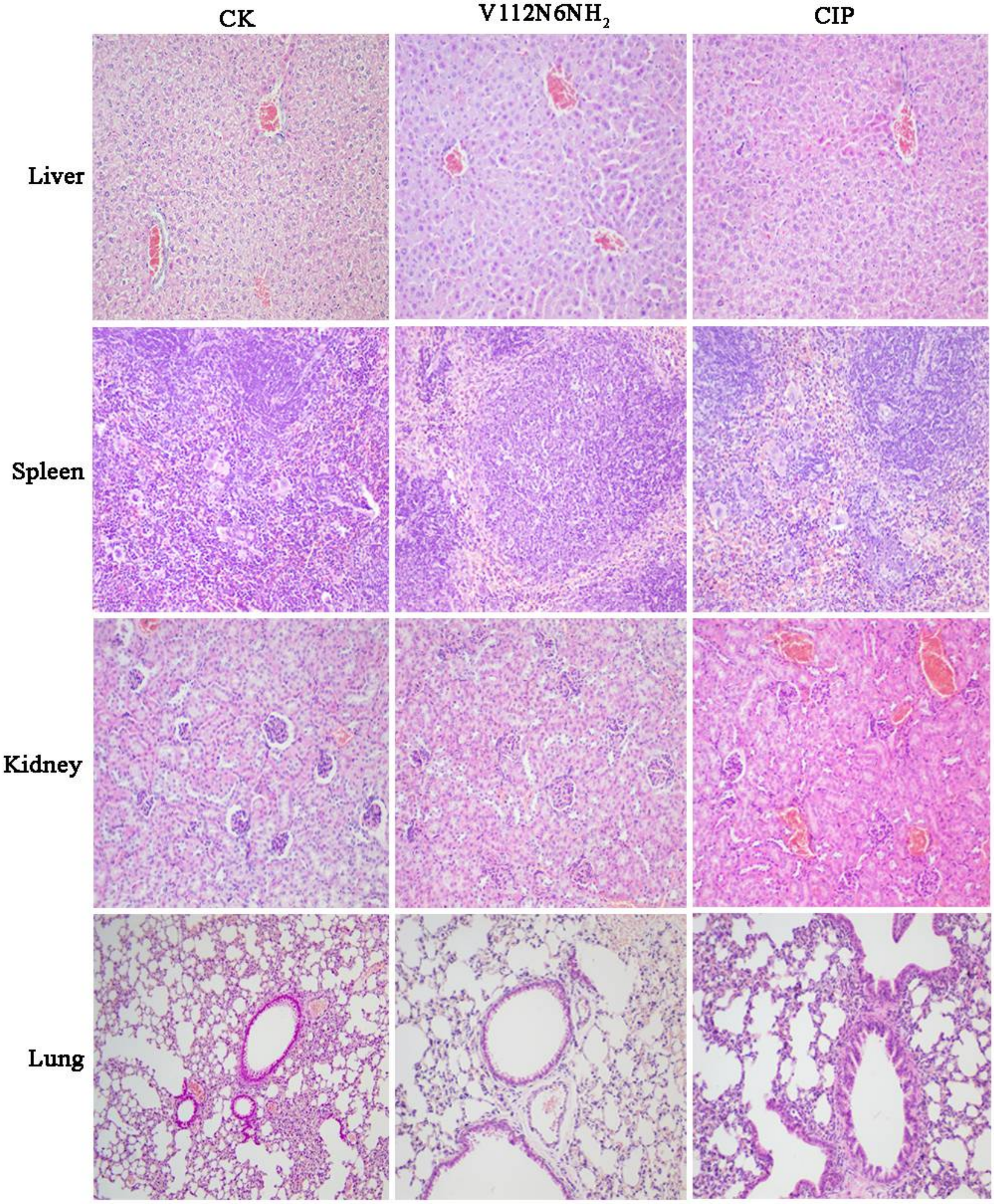

Figure S12-3. Effects of $N6NH_2$ and its analogues on organ injury in mice. Mice were infected intraperitoneally with *A. veronii* ACCC61732 (6×10^8 CFU/mL, 200 μL) and treated with $V112N6NH_2$ (5 μmol/kg) and CIP (1 μmol/kg). The livers, spleens, kidneys and lungs were harvested from mice sacrificed at 24 h after infection.

Table S1. Synergism of $N6NH_2$ analogues and antibiotics.

Combination	Variety	*A. veronii* ACCC61732			
		MIC[a] (μg/mL)	MIC[c] (μg/mL)	FIC	FICI
$N6NH_2$-CIP	$N6NH_2$+CIP	4+0.125	4+0.0078	1+ 0.0625	1.0625
$N6NH_2$-OFX	$N6NH_2$+OFX	4+0.25	4+0.0156	1+ 0.0625	1.0625
$N6NH_2$-NOR	$N6NH_2$ +NOR	4+0.5	4+0.5	1+1	2
$N6NH_2$-ENRO	$N6NH_2$+ENRO	4+0.25	4+0.25	1+1	2
$N6NH_2$-RIF	$N6NH_2$ +RIF	16+0.5	8+0.125	0.5+0.25	0.75[b]
$N6NH_2$-VAN	$N6NH_2$+VAN	16+16	16+1	1+0.0625	1.0625
$N6NH_2$-PMB	$N6NH_2$+PMB	16+8	8+2	0.25+0.25	0.5[a]
$N6NH_2$-STRE	$N6NH_2$+STRE	16+64	8+32	0.5+0.5	1[b]
$N6NH_2$-DOXY	$N6NH_2$+DOXY	16+1	8+0.5	0.5+0.5	1[b]
$N6NH_2$-KANA	$N6NH_2$+KANA	16+8	8+0.5	0.5+0.125	0.625[b]
$N6NH_2$-CHLO	$N6NH_2$+CHLO	16+0.25	16+0.0156	1+0.625	1.0625

Note: MIC[a] indicates the MIC of drug used alone; MIC[c] indicates the MIC of drug used in combination; [a]Synergic effect.; [b]Additive effect; CIP: ciprofloxacin; OFX: ofloxacin; NOR: norfloxacin; ENRO: enrofloxacin; RIF: rifampicin; VAN: vancomycin; PMB: polymyxin B; STRE: streptomycin sulfate; DOXY: doxycycline hyclate; KANA: kanamycin sulfate; CHLO: chloramphenicol.

Table S2. Synergism of $DN6NH_2$ and antibiotics.

Combination	Variety	*A. veronii* ACCC61732			
		MIC[a] (μg/mL)	MIC[c] (μg/mL)	FIC	FICI
$DN6NH_2$-CIP	$DN6NH_2$+CIP	4+0.125	4+0.0078	1+0.0625	1.0625
$DN6NH_2$-OFX	$DN6NH_2$+OFX	4+0.25	4+0.0078	1+0.0625	1.0625
$DN6NH_2$-NOR	$DN6NH_2$ +NOR	4+0.5	2+0.0625	0.5+0.125	0.625[b]
$DN6NH_2$-ENRO	$DN6NH_2$+ENRO	4+0.25	1+0.25	0.25+1	1.25
$DN6NH_2$-RIF	$DN6NH_2$ +RIF	4+0.5	2+0.25	0.5+0.5	1[b]
$DN6NH_2$-VAN	$DN6NH_2$+VAN	4+16	4+1	1+0.0625	1.0625
$DN6NH_2$-PMB	$DN6NH_2$+PMB	4+8	4+0.5	1+0.0625	1.0625
$DN6NH_2$-STRE	$DN6NH_2$+STRE	4+64	2+8	0.5+0.125	0.625[b]
$DN6NH_2$-DOXY	$DN6NH_2$+DOXY	4+1	4+0.0625	1+0.0625	1.0625
$DN6NH_2$-KANA	$DN6NH_2$+KANA	4+8	2+1	0.5+0.125	0.625[b]
$DN6NH_2$-CHLO	$DN6NH_2$+CHLO	4+0.25	4+0.0156	1+0.625	1.0625

Note: MIC[a] indicates the MIC of drug used alone; MIC[c] indicates the MIC of drug used in combination; [b]Additive effect; CIP: ciprofloxacin; OFX: ofloxacin; NOR: norfloxacin; ENRO: enrofloxacin; RIF: rifampicin; VAN: vancomycin; PMB: polymyxin B; STRE: streptomycin sulfate; DOXY: doxycycline hyclate; KANA: kanamycin sulfate; CHLO: chloramphenicol.

Table S3. Synergism of $N6PNH_2$ and antibiotics.

Combination	Variety	*A. veronii* ACCC61732			
		MIC[a] (μg/mL)	MIC[c] (μg/mL)	FIC	FICI
$N6PNH_2$-CIP	$N6PNH_2$+CIP	16+0.125	16+0.0078	1+0.0625	1.0625
$N6PNH_2$-OFX	$N6PNH_2$+OFX	16+0.25	16+0.0156	1+0.0625	1.0625
$N6PNH_2$-NOR	$N6PNH_2$ +NOR	16+0.5	16+0.0313	1+0.0625	1.0625
$N6PNH_2$-ENRO	$N6PNH_2$+ENRO	16+0.25	16+0.0156	1+0.0625	1.0625
$N6PNH_2$-RIF	$N6PNH_2$ +RIF	16+0.5	8+0.25	0.5+0.5	1[b]
$N6PNH_2$-VAN	$N6PNH_2$+VAN	16+16	16+1	1+0.0625	1.0625
$N6PNH_2$-PMB	$N6PNH_2$+PMB	16+8	8+4	0.5+0.5	1[b]
$N6PNH_2$-STRE	$N6PNH_2$+STRE	16+64	16+4	1+0.0625	1.0625
$N6PNH_2$-DOXY	$N6PNH_2$+DOXY	16+1	16+0.0625	1+0.0625	1.0625
$N6PNH_2$-KANA	$N6PNH_2$+KANA	16+8	16+0.5	1+0.0625	1.0625
$N6PNH_2$-CHLO	$N6PNH_2$+CHLO	16+0.25	16+0.0156	1+0.625	1.0625

Note: MIC[a] indicates the MIC of drug used alone; MIC[c] indicates the MIC of drug used in combination; [b]Additive effect; CIP: ciprofloxacin; OFX: ofloxacin; NOR: norfloxacin; ENRO: enrofloxacin; RIF: rifampicin; VAN: vancomycin; PMB: polymyxin B; STRE: streptomycin sulfate; DOXY: doxycycline hyclate; KANA: kanamycin sulfate; CHLO: chloramphenicol.

Table S4. Synergism of $V112N6NH_2$ and antibiotics.

Combination	Variety	*A. veronii* ACCC61732			
		MIC[a] (μg/mL)	MIC[c] (μg/mL)	FIC	FICI
$V112N6NH_2$-CIP	$V112N6NH_2$+CIP	16+0.125	16+0.0078	1+0.0625	1.0625
$V112N6NH_2$-OFX	$V112N6NH_2$+OFX	16+0.25	16+0.0156	1+0.0625	1.0625
$V112N6NH_2$-NOR	$V112N6NH_2$ +NOR	16+0.5	16+0.0625	1+0.125	1.125
$V112N6NH_2$-ENRO	$V112N6NH_2$+ENRO	16+0.25	16+0.0156	1+0.0625	1.0625
$V112N6NH_2$-RIF	$V112N6NH_2$ +RIF	16+0.0625	16+0.0313	1+0.0625	1.0625
$V112N6NH_2$-VAN	$V112N6NH_2$+VAN	16+16	16+1	1+0.0625	1.0625
$V112N6NH_2$-PMB	$V112N6NH_2$+PMB	16+8	1+4	0.0625+0.5	0.5625[b]
$V112N6NH_2$-STRE	$V112N6NH_2$+STRE	16+64	16+4	1+0.0625	1.0625
$V112N6NH_2$-DOXY	$V112N6NH_2$+DOXY	16+1	16+0.0625	1+0.0625	1.0625
$V112N6NH_2$-KANA	$V112N6NH_2$+KANA	16+8	16+0.5	1+0.0625	1.0625
$V112N6NH_2$-CHLO	$V112N6NH_2$+CHLO	16+0.25	16+0.0156	1+0.0156	1.0625

Note: MIC[a] indicates the MIC of drug used alone; MIC[c] indicates the MIC of drug used in combination; [b]Additive effect; CIP: ciprofloxacin; OFX: ofloxacin; NOR: norfloxacin; ENRO: enrofloxacin; RIF: rifampicin; VAN: vancomycin; PMB: polymyxin B; STRE: streptomycin sulfate; DOXY: doxycycline hyclate; KANA: kanamycin sulfate; CHLO: chloramphenicol.

Table S5. Synergism of Guo-$N6NH_2$ and antibiotics.

Combination	Variety	*A. veronii* ACCC61732			
		MIC[a] (µg/mL)	MIC[c] (µg/mL)	FIC	FICI
Guo-$N6NH_2$-CIP	Guo-$N6NH_2$+CIP	8+0.125	8+0.0078	1+0.0625	1.0625
Guo-$N6NH_2$-OFX	Guo-$N6NH_2$+OFX	8+0.25	8+0.0156	1+0.0625	1.0625
Guo-$N6NH_2$-NOR	Guo-$N6NH_2$ +NOR	8+0.5	4+0.25	0.5+0.5	1[b]
Guo-$N6NH_2$-ENRO	Guo-$N6NH_2$+ENRO	8+0.25	8+0.0156	1+ 0.0625	1.0625
Guo-$N6NH_2$-RIF	Guo-$N6NH_2$ +RIF	16+0.5	8+0.25	0.5+0.5	1[b]
Guo-$N6NH_2$-VAN	Guo-$N6NH_2$+VAN	16+16	16+1	1+0.0625	1.0625
Guo-$N6NH_2$-PMB	Guo-$N6NH_2$+PMB	16+8	4+2	0.25+0.25	0.5[a]
Guo-$N6NH_2$-STRE	Guo-$N6NH_2$+STRE	16+64	8+32	0.5+ 0.5	1[b]
Guo-$N6NH_2$-DOXY	Guo-$N6NH_2$+DOXY	16+1	16+0.0625	1+ 0.0625	1.0625
Guo-$N6NH_2$-KANA	Guo-$N6NH_2$+KANA	16+8	16+0.5	1+0.0625	1.0625
Guo-$N6NH_2$-CHLO	Guo-$N6NH_2$+CHLO	16+0.25	16+0.0156	1+ 0.625	1.0625

Note: MIC[a] indicates the MIC of drug used alone; MIC[c] indicates the MIC of drug used in combination; [a]Synergic effect.; [b]Additive effect; CIP: ciprofloxacin; OFX: ofloxacin; NOR: norfloxacin; ENRO: enrofloxacin; RIF: rifampicin; VAN: vancomycin; PMB: polymyxin B; STRE: streptomycin sulfate; DOXY: doxycycline hyclate; KANA: kanamycin sulfate; CHLO: chloramphenicol.

Table S6. MIC values of $N6NH_2$ and its analogues against *A. veronii* ACCC61732 under different conditions.

Peptide	Control[a]	Temperature (°C)						pH value					Physiological salt (mM)						Enzyme		
		4	20	40	60	80	100	2	4	6	8	10	50	100	200	300	400	500	Pepsin	Trypsin	Proteinase K
$N6NH_2$	4	4	4	4	4	4	8	4	4	4	4	4	4	4	8	8	8	8	4	> 128	> 128
$DN6NH_2$	4	4	4	4	4	4	8	2	2	4	4	4	4	4	4	4	4	4	4	4	4
$N6PNH_2$	16	16	16	16	16	16	32	8	16	16	8	8	8	8	16	16	16	32	16	> 128	> 128
$V112N6NH_2$	16	16	16	16	16	16	32	16	16	16	16	8	8	16	16	16	32	32	16	> 128	> 128
Guo-$N6NH_2$	8	8	8	8	8	8	16	8	8	8	4	4	8	8	8	8	8	16	8	> 128	> 128

Note: [a] The control MIC values were determined in the absence of these temperature, acid and alkali, physiological salt, and enzyme condition.

Table S7. Effects of $N6NH_2$ and its analogues on inner membrane of *A. veronii* ACCC61732.

Peptides	Time (h)	Permeability (%)		
		1 × MIC	2 × MIC	4 × MIC
$N6NH_2$	0.5	0	0	0
	2	1.39	1.49	1.12
$DN6NH_2$	0.5	0	9.16	41.93
	2	3.42	9.83	33.73
$N6PNH_2$	0.5	0	0.68	0
	2	2.11	1.35	1.23
V112N6NH2	0.5	0	0	4.28
	2	1.8	5.67	23.93
Guo-N6NH2	0.5	0.63	0.55	0
	2	4.96	1.91	2.16
CIP	0.5	2.85	4.09	2.55
	2	0.78	1.19	4.43

Table S8. Primers of the *vacJ* gene.

Primer	Sequence (5'→3')	Product (bp)
*vacJ*F	AGTGGTACATGCCTATGC	482
*vacJ*R	GGTAGAAGTCCTTGGTCAG	
